# What Are the Net Benefits of Reducing the Ozone Standard to 65 ppb? An Alternative Analysis

**DOI:** 10.3390/ijerph15081586

**Published:** 2018-07-26

**Authors:** Sabine S. Lange, Sean E. Mulholland, Michael E. Honeycutt

**Affiliations:** 1 Toxicology Division, Texas Commission on Environmental Quality, P.O. Box 13087, MC-168, Austin, TX 78711, USA; michael.honeycutt@tceq.texas.gov; 2 Department of Economics, Management, and Project Management, West Carolina University, Cullowhee, NC 28723, USA

**Keywords:** ozone, air pollution toxicology, air pollution standards, environmental policy, benefit-cost analysis

## Abstract

In October 2015, the United States Environmental Protection Agency (EPA) lowered the level of the ozone National Ambient Air Quality Standard (NAAQS) from 0.075 ppm to 0.070 ppm (annual 4th highest daily maximum 8-h concentration, averaged over three years). The EPA estimated a 2025 annual national non-California net benefit of $1.5 to $4.5 billion (2011$, 7% discount rate) for a 0.070 ppm standard, and a −$1.0 to $14 billion net benefit for an alternative 0.065 ppm standard. The purpose of this work is to present a combined toxicological and economic assessment of the EPA’s benefit-cost analysis of the 2015 ozone NAAQS. Assessing the quality of the epidemiology studies based on considerations of bias, confounding, chance, integration of evidence, and application of the studies for future population risk estimates, we derived several alternative benefits estimates. We also considered the strengths and weaknesses of the EPA’s cost estimates (e.g., marginal abatement costs), as well as estimates completed by other authors, and provided our own alternative cost estimate. Based on our alternative benefits and cost calculations, we estimated an alternative net benefit of between −$0.3 and $1.8 billion for a 0.070 ppm standard (2011 $, 7% discount rate) and between −$23 and −$17 billion for a 0.065 ppm standard. This work demonstrates that alternative reasonable assumptions can generate very difference cost and benefits estimates that may impact how policy makers view the outcomes of a major rule.

## 1. Introduction

### 1.1. The National Ambient Air Quality Standards

The U.S. Environmental Protection Agency (EPA) regulates 6 air pollutants (called “criteria pollutants”) through the National Ambient Air Quality Standards (NAAQS) program: ozone, particulate matter (PM), carbon monoxide (CO), lead, nitrogen dioxide (NO_2_), and sulfur dioxide (SO_2_). Section 109 of the Federal Clean Air Act (FCAA) directs the EPA Administrator to set NAAQS that are requisite to protect public health with an adequate margin of safety. The NAAQS have four equally important elements: the indicator, the level, the averaging time, and the statistical form.

The NAAQS review involves multiple steps and documents (the history of the NAAQS process is reviewed in Bachmann (2007) [1]). EPA staff complete a science assessment, an exposure assessment, and a risk assessment of the criteria pollutant, then write a recommendation to the EPA administrator about the adequacy of the current indicator, level, averaging time, and form of the NAAQS for that pollutant. The EPA administrator then proposes a rule that would maintain or change the standard, and if warranted a cost-benefit analysis of the rule is conducted in the form of a regulatory impact analysis (RIA). Although the EPA administrator is not legally allowed to consider cost when setting a NAAQS (Whitman vs. American Trucking Association, 2001), the RIA provides information to the public about the approximate costs and benefits of the proposed rule, and RIAs are required to comply with executive orders 12866 [2] and 13563 [3]. Based on the scientific data, analysis, and uncertainty, the EPA Administrator must make a policy decision about where to set the level, averaging time, and form of the NAAQS. The science cannot and does not dictate a specific policy outcome, but it is instrumental in choosing and justifying the choices made in setting the NAAQS.

### 1.2. Ozone

Ozone (chemical name: O_3_) is a chemical that is found both in the stratosphere, where it forms the protective ozone layer, and in the troposphere (ground level), where it contributes to the air pollution known as smog. Ozone is primarily an outdoor pollutant [4,5,6,7,8]. Ground level ozone is formed when precursor emissions (nitrogen oxides (NO_x_) and volatile organic compounds (VOCs) released from sources such as automobile exhaust, power plant emissions, and wildfires) react with sunlight. In most of the U.S., the level of NO_x_ is the limiting factor for ozone formation, not VOCs. Therefore, in the EPA’s RIA for the current ozone rule, attainment of the ozone standard is modeled by a reduction of NO_x_ precursor emissions, and not from VOC reduction [9,10]. A comprehensive review of ozone chemistry is included in Section 3 of the U.S. EPA’s 2013 Integrated Science Assessment [11].

Ozone is a focus of air pollution regulations because it is a highly reactive oxidizing agent that can cause damage to tissues when inhaled. Human controlled exposure experiments have shown that ozone can diminish lung function when the total ozone dose is above a certain threshold [12,13]. The EPA Administrator placed the most weight on human controlled exposure studies for the 2015 ozone review, and these studies formed the basis for the EPA’s choice of standard levels [14,15]. A large literature of epidemiology studies also exists that investigate the associations between health effects in populations and ambient ozone concentrations [16,17,18,19,20,21,22,23]. These studies are useful, but can only provide information about correlation between ozone and health effects, not causation [24]. The EPA administrator placed far less weight on epidemiology studies in the review of the ozone NAAQS because of significant uncertainties in their conclusions [14,15]. However, the EPA used results from epidemiology studies when estimating the health benefits attributable to decreasing the ozone standard [9,10], and as such these studies form the basis of our discussion of ozone attributable health benefits below.

### 1.3. The Ozone NAAQS Cost and Benefit Analysis

In October 2015, the EPA lowered the level of the current primary and secondary ozone standards (last set in 2008) from 0.075 parts per million (ppm; annual fourth highest daily maximum 8-h concentration, averaged over 3 years) to 0.070 ppm [15]. In their draft RIA released with the proposed rule [9], and their final RIA released with the final rule [10], the EPA estimated the 2025 national, non-California net benefits for the 0.070 ppm standard (ranging from $1.5 to $9.1 billion) and the 0.065 ppm standard (ranging from −$1 to $23 billion; Supplemental Table S1). Also reported are post-2025 California net benefits.

The EPA asserts that lower levels of ozone, as well as ancillary decreases in fine particulate matter (PM_2.5_), will improve public health by reducing incidence of morbidity and premature mortality. The avoided morbidity and mortality are calculated using concentration-response (C-R) functions from various epidemiology studies that measured the association between ozone or PM_2.5_ and health effects, and are then monetized to calculate total benefits. The costs are based on illustrative control strategies across the U.S. (actual costs may be different from EPA’s estimate because each state will derive their own plan for how to attain the ozone standard). For details on how these benefits and costs were calculated, refer to the EPA’s RIAs [9,10]. The costs and benefits in the ozone RIA, while technically irrelevant to the standard-setting process, have been discussed at length by proponents and challengers of the rule alike, and so deserve an in-depth analysis to assess the accuracy of the conclusions.

The following is an analysis of the underlying assumptions of EPA’s benefits and costs estimates, conducted to determine the dependence of the estimates on these assumptions. We present considerations for assessing the quality of the epidemiology studies that underlie the benefits estimates, and alternative methods for calculating the costs. Our alternative benefits and costs calculations are based both on the draft RIA and the final RIA benefit and cost estimates. We used both RIAs so that we could compare our methodology with other studies as well as with the EPA’s final 2015 rule. Moreover, our primary discussion focuses on the costs and benefits of attaining a 0.065 ppm standard (and not the actual 0.070 ppm standard), because many other cost analyses provided estimates for 0.065 ppm. We also provide estimates of the costs and benefits of a 0.070 ppm standard.

## 2. Assessment of Health Effect Outcomes Attributed to Ozone

The focus of this part of the analysis will be on our confidence in the epidemiology studies used to quantify benefits in the EPA’s ozone RIAs. When an association between an air pollutant and a health effect is found in an epidemiology study, it could be due to confounding, bias, chance, or causation [24]. Once confounding, bias, and chance have been considered, it is important to integrate the epidemiological evidence with information from other types of studies (animal experiments, human controlled exposure experiments, etc.) to determine whether causation is a plausible reason for the association. Finally, when used for a risk assessment, the information from the epidemiology study must be examined to determine whether the study can be used to quantify future risk (i.e., that future populations will be at risk from this agent [25]). If this evidence all together suggests a causal link between ozone and the health effect, then the EPA is justified in monetizing the benefits from reducing the cause (i.e., ozone). If there is no causal link, then the EPA is not justified in monetizing benefits from reducing ozone, because no benefits would ensue. Below we discuss some key considerations that address bias (exposure measurement error), confounding (copollutants), chance (regional heterogeneity), integration of evidence (thresholds, coherence), and future risk (recent data). We present an example system for scoring these considerations, and then, using this information, offer an alternative method for calculating benefits.

### 2.1. Considerations for Air Pollution Epidemiology Studies

#### 2.1.1. Exposure Measurement Error

Ideally, to accurately estimate the association between air pollution and health effects, researchers would prefer personal exposure data, i.e., measures of the total inhaled dose of an air pollutant that a person inhales over a specified time period. However, most air pollution epidemiology studies do not have this information, and instead use stationary ambient monitors to measure the concentrations of air pollutants to which nearby (and not so nearby) populations may be exposed [16,18,19,21,26,27]. These studies then assume that the measurements from these monitoring stations are reasonable approximations of what a nearby resident inhales. Inaccurate exposure measurement like this causes exposure measurement error in the epidemiology estimates [28,29]. This is a major concern in ozone epidemiology study results because various studies have found that personal ozone concentrations are lower (by up to 90%) than ambient ozone concentrations [6,7,8], and that ambient ozone concentrations cannot necessarily be used to predict personal concentrations [7,8]. Because of the complexities of epidemiology statistical models and the presence of other errors, the estimated effect of ozone on health could either be an underestimate of the true effect, or it could falsely report an effect that is not actually due to ozone concentrations [28,30,31].

Another consideration for ozone exposure is how well the concentration represents a person’s total inhaled dose. Many human controlled exposure studies have been conducted that expose people to known concentrations and known doses of ozone (total dose being a combination of concentration, duration of exposure, and ventilation rate [32,33,34]). By considering only concentration and failing to account for duration of exposure and ventilation rate (i.e., exercise level), a person’s total inhaled dose of ozone is misrepresented and, more importantly, so are the resulting potential effects of the exposure.

Here we applied a confidence rating to epidemiology studies based on how well they consider exposure measurement error. We applied this type of confidence assessment to all the considerations discussed in this report, and then provide examples of how these can be applied to actual studies. An epidemiology study with a low confidence in exposure measurement error would have used ambient monitors in the individual’s county, zip code, or entire metropolitan area to represent exposure, with no consideration of personal exposure or exposure measurement error. A medium confidence would be indicated if modeling was done to better estimate the ambient concentration closer to the individual’s home or work (e.g., using atmospheric chemistry and meteorology to provide a better estimate of pollutant concentrations), or if statistical tests were applied that take into consideration the exposure measurement error [31,35,36]. A high confidence would be indicated if personal concentrations and some consideration of dose (i.e., duration and ventilation rate) had also been taken into account when judging exposure.

#### 2.1.2. Confounding and Copollutants

**Figure 1:**
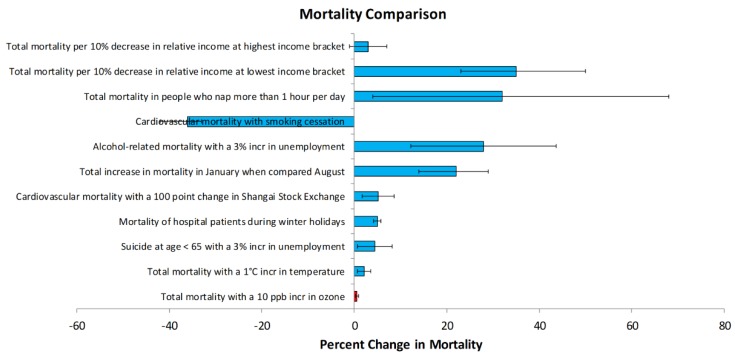
Mortality attributed to ozone, compared to other factors that contribute to mortality [38,39,40,41,42,43,44].

Confounders are factors that are correlated both with the health effect and the pollutant of interest, and that could be the actual cause of a health effect. Confounders need to be ruled out to be confident that the health effect is actually being caused by the pollutant [24]. Confounding is even more likely when the correlation between the health effect and the pollutant is not very strong (e.g., an increased risk of a health effect of <1.5), as is commonly seen with ozone epidemiology studies [16,26]. Low effect estimates are more vulnerable because they can be generated by residual and/or unmeasured confounding, even in the absence of a genuine causal association [37]. Some of the important confounders to consider in ozone epidemiology studies are other pollutants such as PM, pollen (especially when assessing asthma), temperature (because both ozone and temperature increase during the day and during the summer, and temperature is linked to morbidity and mortality), smoking status, socioeconomic status, pre-existing medical conditions, etc. [38,39,40,41,42,43]. In fact, many of these factors affect morbidity and mortality far more than ozone (Figure 1). As with exposure measurement error, confounding can bias effect estimates so that they are lower than the actual association, or higher than the actual association, depending on the circumstances [37].

We assigned a low confidence to any epidemiology study that failed to consider any confounders or copollutants in its assessment of the correlation between ozone and a health endpoint. A medium confidence study would have investigated several confounders or copollutants in the statistical association between ozone and the health endpoint. A high confidence study would look at many confounders and copollutants and would use models to attempt to control for residual confounding (this is measurement error within the confounding variables) and unmeasured confounding (confounders that were not considered in the model).

#### 2.1.3. Regional Heterogeneity

**Figure 2:**
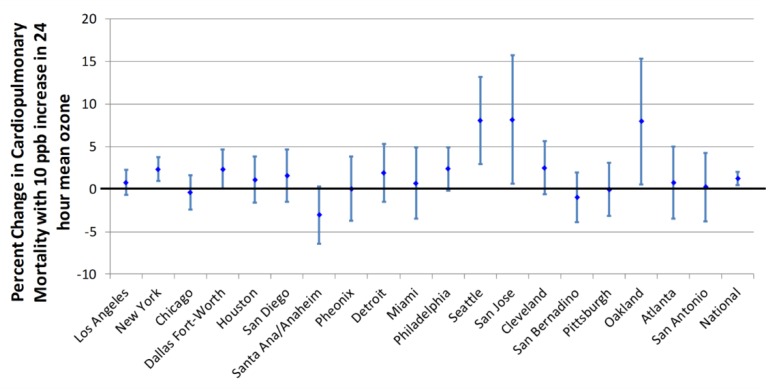
Regional heterogeneity in cardiopulmonary mortality estimates from Huang et al. (2005) [45]. Estimates whose error bars cross the x-axis at zero (thick black line) are not statistically significant. The highest mean daily ozone levels in these cities for this analysis were in San Bernadino, San Diego, Los Angeles, Phoenix, and Dallas-Fort Worth. The lowest mean ozone levels were in Oakland, Miami, San Jose, Houston, and Seattle.

Regional heterogeneity occurs when different cities or regions show different associations between a pollutant and the health effect. Regional heterogeneity makes it difficult to decide which association to choose for a health effects assessment like the EPA’s RIAs [9,10]. When regional heterogeneity cannot be explained (i.e., there are no variables included in the study that can explain why a pollutant would cause an effect in one city but not another), then it casts doubt on the causal association between the pollutant and the claimed health effect [16]. For regional heterogeneity to be measured, a study must assess multiple cities simultaneously, using the same methodologies and data sources. If multiple studies using similar methods have investigated the correlation between the pollutant and the health effect in multiple cities, it allows the comparison of whether different studies consistently find the same results in the same cities, or find different results in the same cities. Regional heterogeneity is an oft-observed characteristic of the associations between ozone and morbidity or mortality (Figure 2) [16,21,26,45].

We assigned an epidemiology study a low confidence for regional heterogeneity if it was a single-city study; if it was a multi-city study and did not report the association by individual cities; or if it was a part of a set of studies showing inconsistent results in the same cities over the same time periods. A medium confidence would be given if regional/city specific associations were presented and regional heterogeneity was observed; or if a set of studies showed consistent results in the same cities over the same time periods. A high confidence would be given if regional/city specific associations were presented and there was no regional heterogeneity (i.e., there was regional homogeneity); or a plausible, evidence-based explanation or analysis for the observed regional heterogeneity was provided.

#### 2.1.4. Consistency

Causal conclusions are strengthened when many independent studies observe a statistically significant association between a potential cause and a health effect [46]. In particular, different types of studies that show similar results will strengthen the conclusion. However, a set of studies provide much weaker evidence for consistency if all the studies suffer from the same error or bias, for example the exposure measurement error discussed above. The source and quality of the data, and the sample size, must also be considered: if all the studies that show the same association used data from the same source and during the same time period, then the studies are not independent (they are just reanalyzing data), and it would cast doubts on their conclusions if they did not have consistent results. Another important consideration is the presence of publication bias [24]. Publication bias is caused by the selective publication of studies showing significant associations (either positive or negative) relative to those that show no association. For example, several recent publications looked for and found evidence of publication bias in ozone short-term mortality epidemiology studies [47,48,49].

In this case, we used the literature as a whole (all of the studies that address a particular association between a pollutant and a health effect) to assess consistency between the health effect and the pollutant. We assigned a low confidence for consistency if only one study has been performed (and therefore consistency could not be assessed), or fewer than half of the studies showed consistent results. A medium confidence would be given if more than half of the study results were consistent with one another, but there was evidence of publication bias or publication bias had not been assessed; if the studies were all based on the same dataset; or if they all suffered from the same systematic error or bias (e.g., exposure measurement error). A high confidence would be given if more than half of the studies in the literature had consistent results; and there is a demonstrated lack of publication bias in the literature; and the results were derived from independent datasets; and the studies with consistent results did not all have the same systematic error or bias.

#### 2.1.5. Consideration of Thresholds

When assessing risk from a pollutant, it is important to study and understand whether the pollutant demonstrates a threshold in its health effects. That is, is there a dose or concentration below which no health effects are observed? The lowest dose or concentration at which a health effect is observed (typically in a controlled experiment) can be used as the threshold for that pollutant [50]. Animal and human toxicology studies strongly support the existence of a threshold for ozone-mediated health effects [11]. Confidence in the threshold for a pollutant is strengthened by the identification of the mode of action (MOA) by which the pollutant harms the body [51,52]. For example, the MOA for ozone is known; upon inhalation, antioxidants in the fluids lining the respiratory tract react with the ozone, preventing harm. However, if too much ozone is inhaled (i.e., the amount of ozone is greater than a certain threshold), it can overwhelm the antioxidant defenses and cause inflammation and damage to the lungs, decreased lung function, and increased airway sensitivity in asthmatics (reviewed in a previous study [11], Chapter 5). Some epidemiology studies do not consider thresholds of effect in their models, and instead project that health effects will occur at any concentration above zero [18,19,22,26], which can overestimate the health benefits of decreasing ambient concentrations of a pollutant with a threshold. Therefore, it is important for a threshold to be considered in epidemiological modeling, if evidence supports the presence of a threshold. Of note, bias and error in the data used in an epidemiology study may obscure the presence of a threshold, meaning that many epidemiology studies may not be capable of identifying a threshold, even if one exists [51,53].

We assigned a low confidence for consideration of thresholds if the study assumed that no threshold was present when there is other evidence that a threshold may exist (e.g., from toxicology or human experimental studies). A medium confidence would be assigned if a study used a modeling approach that allowed for the possibility of a threshold (e.g., a linear model with splines), but if the study suffered from exposure measurement error or other biases that are known to obscure thresholds even if a threshold is actually present in the data [51,53]. A high confidence would be given if the study modeled the pollutant-health effect association with a threshold when there is evidence that one exists and provided results using the threshold model.

#### 2.1.6. Coherence

**Figure 3:**
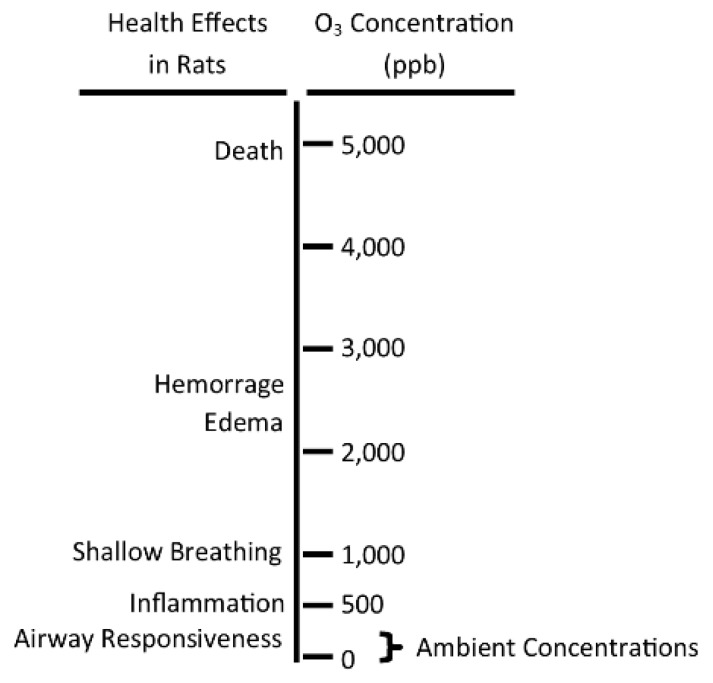
Health effects of rats exposed to different concentrations of ozone. With exposure of rats to increasing concentrations of ozone (single exposure for ~8 h while at rest), there is an increasing severity of response. The range of ambient ozone concentrations is noted at the bottom (modified from a previous study [55] and references therein).

When assessing a study, it is important to consider the coherence of the findings with other scientific studies, particularly studies from other branches of science [54]. Animal and human toxicology studies can be used to assess whether the results from epidemiology studies are plausible (called biological plausibility). For example, Figure 3 presents ozone concentrations at which various health effects occur in rats upon exposure to ozone (modified from a previous study [55]). At higher ozone concentrations well above those found in ambient air (~5 ppm) severe responses like hemorrhaging and death take place. At lower concentrations (~0.05 ppm), mild responses like airway responsiveness occur, but more severe responses such as hemorrhaging and death are not present. The range of daily ambient ozone concentrations (extends from zero up to ~0.15 ppm [56]) is noted in the figure. Therefore, an epidemiology study showing associations between ambient concentrations of ozone and mild respiratory health effects is biologically plausible and consistent with ozone’s MOA (discussed in Section 2.1.5). However, some epidemiology studies report the relationship between severe health effects, such as death, and ozone at ambient concentrations (e.g., Smith et al. (2009), Zanobetti & Schwartz (2008) [16,26]) even though human and animal toxicology studies find no such association (Figure 3), and so these health effects are not biologically plausible.

Another important consideration is the amount of exposure that the population is expected to have to the pollutant. If there is no expectation of exposure to a pollutant, then there can be no expectation of a health effect caused by that pollutant. As noted above, ozone is an outdoor pollutant [4,5,6,7,8], so for the population to be exposed to significant concentrations, they have to be outdoors.

We assigned a low confidence for coherence if the study suggested that a health effect occurred that is either a completely different effect that is not supported by the MOA (e.g., an inhalation exposure causing toe fungus); or that occurs at far different concentrations; or if the population was unlikely to have had a significant exposure to the pollutant. A medium confidence would be given if the observed health effect could be extrapolated to be supported by the MOA, or if there was at least a fair possibility that the population was exposed to significant amounts of the pollutant. For example, if the health effect was consistent with the pollutants MOA, but was a more severe response than expected (e.g., respiratory hospitalizations upon ozone exposure), or if a more severe response was associated with a population that was likely to be exposed (e.g., outdoor workers exposed to ozone). A high confidence would be given if the health effect was entirely supported by a plausible MOA, the magnitude of effect was consistent with the appropriate concentrations for the pollutant, and the population was plausibly exposed to significant amounts of the pollutant.

#### 2.1.7. Recent Data

**Figure 4:**
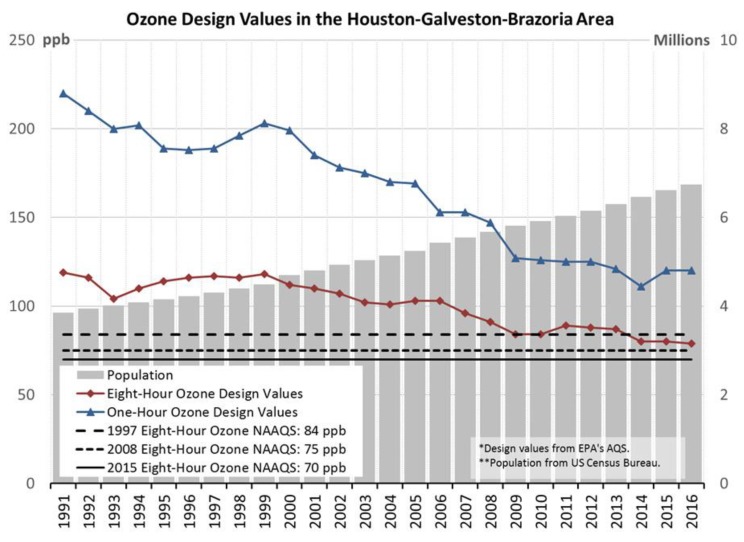
Ozone regulatory design values in the Houston-Galveston-Brazoria area of Texas from 1991 to 2016. Ozone design values are regulatory values that comprise the annual fourth-highest daily maximum 8-h ozone concentration, averaged over 3 years (eight-hour ozone design values), or the maximum hourly average concentration (one-hour ozone design values). Texas Ozone Data: http://www.tceq.texas.gov/airquality/airsuccess/airSuccessCriteria.

Levels of air pollution have decreased drastically in the U.S. since the FCAA was voted into law in 1970 [1] (Figure 4 provides an example of decreases in ozone concentrations in Houston, Texas from 1991 to 2016). Because of the decrease in air pollution and the concept of concentration-response, health effects that may have been caused by air pollution in the 1970’s are likely to be more severe than the effects potentially caused by current, lower ambient concentrations. Therefore, using more recent data is important to determine if the ambient concentrations found currently could be causing the measured health effects.

If more than half of a study’s pollution and health effects data were older than 20 years (i.e., before 1995) then we assigned it a low confidence value. If more than half of a study’s pollution and health effects data were older than 10 years (i.e., up to 2005) it was assigned a medium confidence value. A study assigned a high confidence value would have more than half of its pollution and health effects data from less than 10 years ago (i.e., 2006–2015).

## 3. Assessment of Health Effects Studies in the EPA RIA

The EPA applied selection criteria to identify the epidemiology studies to be used for quantification of health benefits from ozone reduction [9,10]. These inclusion criteria were: preference for studies that were published in the peer-reviewed literature; those with better study designs; those that investigated longer time periods and are more recent; those that calculated summer-only effect estimates; those that specifically investigated the effects of ozone on subpopulations based on age, sex, pre-existing conditions, or other factors; and studies with a large sample size. Often many health effect estimates are presented in a study. The EPA preferentially chose estimates that: came from nationwide U.S. data; *D* as a copollutant; measured economically valuable health effects; and did not include overlapping health endpoints (to avoid double counting).

Based on these inclusion criteria and preferences, 16 studies were selected by the EPA to form the foundation of their ozone health benefits assessment in the draft RIA [9], and 15 studies were used in the final RIA [10]. In the draft and final RIA, 26 studies were used to quantify cobenefits from the reduction of PM_2.5_. Upon monetization of the benefits from reduction of ozone or PM_2.5_, it became clear that most of the benefits (>95%) were attributable to reduction in mortality. The EPA used Smith et al. (2009) and Zanobetti & Schwartz (2008) to quantify ozone-associated mortality [16,26]; and Krewski et al. (2009) and Lepeule et al. (2012) to quantify PM_2.5_-associated mortality [57,58]. Because these four studies account for most of the benefits, they alone are discussed in the following text, with confidence ratings for each consideration shown in Table 1, and the details of the confidence ratings in Appendix A.

### 3.1. Ozone-Associated Short-Term Mortality

Monetization of short-term mortality provides most of the EPA’s ozone-associated benefits from decreasing the ozone standard. Short-term mortality refers to the association between all non-accidental causes of mortality and short exposures to ozone (1–7 days). Effect estimates from Smith et al. (2009) [16] and Zanobetti & Schwartz (2008) [26] were used to estimate benefits from reductions in ozone-attributed mortality. By our considerations, Smith et al. (2009) produced a higher quality study than Zanobetti & Schwartz (2008) (Table 1, Appendix A). Smith et al. (2009) concluded that there was, on average, a 0.4% increase in short-term, all-cause non-accidental mortality with a 10 ppb increase in daily 8-h maximum ozone concentrations. However, we still have major concerns with exposure measurement error, regional heterogeneity, and lack of biological plausibility in this study. The authors themselves stated:

“We caution, again, that any national summary, even a population-weighted average, will conceal the still-unexplained heterogeneities. Further, we believe that the heterogeneity and sensitivity of ozone effect estimates to a variety of covariates leaves open the issue of whether or not ozone is causally related to mortality.”

([16], p. 54)

### 3.2. Mortality from Particulate Matter

About 75% of the benefits from lowering the ozone standard were derived from a predicted coincident decrease of PM_2.5_ [9,10]. Long-term exposure to PM_2.5_ has been associated with premature mortality [57,58]. PM is a mixture of many types of small particles, however the reductions in NO_x_ required to decrease ozone concentrations can only be predicted to decrease *nitrate* PM_2.5_. Toxicological studies have exposed experimental animals to particulate ammonium or sodium nitrate, but have found very few physiological effects (reviewed in several papers [59,60,61,62]), even at levels in the range of mg/m^3^ (the level of the annual PM_2.5_ standard is 12 μg/m^3^ [63]). These studies have examined a variety of endpoints, including lung function and morphology, cardiac function, local and systemic inflammation, and resistance to infection, however there is no clear evidence that nitrate components of PM_2.5_ adversely impact these endpoints. Similarly, human controlled exposure studies have exposed people to nitrate PM_2.5_ at concentrations ranging from 200–7000 μg/m^3^, and no significant pulmonary effects have been observed in healthy [64,65,66] or asthmatic [64,66] subjects. Those epidemiological studies that have investigated the association between nitrate PM_2.5_ and mortality or morbidity have found mixed and conflicting results [67,68,69,70]. Altogether, data for nitrate PM_2.5_ demonstrate very little evidence of toxicity, particularly at ambient levels [59,60,61,62,71]. This calls into question EPA’s estimate that a decrease in PM_2.5_ caused by lowering NO_x_ concentrations would reduce mortality, which adds a significant layer of uncertainty to the EPA’s monetized benefits concerning PM_2.5_.

Based on our assessment, Krewski et al. (2009) [57] and LePeule et al. (2012) [58] produced studies of low and medium confidence, respectively (Table 1, Appendix A). Both studies indicated that there is a statistically significant association between long-term exposure to PM_2.5_ and total mortality. However, these studies suffer from major concerns about exposure measurement error (PM_2.5_ is only measured in a few years of the study and is extrapolated to the rest of the study), regional heterogeneity (multiple cities are investigated but are not reported), and the biological plausibility of the causal association (there is little evidence from toxicology or human studies that ambient concentrations of PM_2.5_ are lethal, and particularly not the nitrate PM_2.5_ that is most relevant to this review).

## 4. Adjusted Benefits Estimates

### 4.1. Benefits Approach #1

Section 2 and Section 3 of this paper reveal significant concerns with the methodology of the studies used to quantify benefits. Ideally, we would generate our own measures of benefits. However, much of the data used by these studies are not publicly available. In the following two sections, we provide two alternative benefits measures that account for the weaknesses in these studies. The first uses our confidence determinations described in the previous sections. The second follows methods developed by Cox (2012) [25]. As shown in Table 5–20 in the draft RIA [9] and in Supplemental Table S2, the EPA estimated that the avoided short-term mortality benefits of lowering the ozone NAAQS from 0.075 ppm to 0.065 ppm are worth $6.4 billion for 2025 using Smith et al. (2009) [16], or are $11 billion using Zanobetti & Schwartz (2008) [26]. Yet, both studies failed to convey a high level of confidence in any of our areas of consideration.

To account for the uncertainties from our seven areas of concern, we assigned probability weights to each confidence: p_L_ = 0.4 for low confidence; p_M_ = 0.75 for medium confidence; and p_H_ = 1.0 for high confidence. For sensitivity, we considered using the EPA’s 95-percent confidence interval measures for ozone exposure. However, the EPA does not provide confidence intervals for the PM_2.5_-related cobenefits. Therefore, we were unable to estimate a 95-percent confidence interval for the combined benefits of ozone and PM_2.5_ reductions. Instead we assigned low corresponding value probability weights of p_LL_ = 0.3, p_ML_ = 0.6, and p_HL_ = 0.9 and high corresponding value probability weights of p_LH_ = 0.5, p_MH_ = 0.9, and p_HH_ = 1.1. Giving each component equal weight in our assessment, we multiplied the probability weight by 0.1419 (or 1/7). We then summed up each of the seven terms to calculate an overall measure of confidence that ranges from 0.4 to 1.0. Therefore, each paper’s overall confidence measure can be described by: $\Sigma_{i=1}^{7}=\;p_{i}\times 0.1429$. We then multiplied the overall confidence measure by the monetized benefits reported by the EPA to generate an alternative measure of benefits that accounts for the confidence in the underlying studies. Table 2 shows the calculation for each of the 7 components for Smith et al. (2009) and the overall confidence measure of 0.600 (range of 0.472 to 0.729). Although the Smith et al. (2009) study earned a medium confidence in the cofounders category, the EPA did not use the model that included PM_10_ as a confounder, and so we used a low confidence in this category. Performing the same exercise for Zanobetti & Schwartz (2008) [26] generates an overall confidence measure of 0.500; for Krewski et al. (2009) [57] our overall confidence measure is 0.500; and for Lepeule et al. (2012) [58] it is 0.600.

To construct our first alternative measure of benefits, we multiplied these overall confidence measures by the EPA’s estimated benefits. Supplemental Table S2 recreates a portion of Tables 5–20 and 5–22 from the draft RIA [9] using our alternative benefits measures. Accounting for our confidence in the methodology used by Smith et al. (2009) [16], we estimated that the annual benefits in 2025 from lowering the ozone standard from 0.075 ppm to 0.065 ppm are closer to $3.8 billion and not the $6.4 billion estimated by the EPA, and for Zanobetti & Schwartz’s (2008) [26] our estimate is $5.5 billion instead of $11 billion. For a 0.065 ppm ozone standard, our alternative benefits for reducing the PM_2.5_ range from $7.2 billion to $14 billion at a 7% discount rate. Combining our alternative benefits from ozone reductions and our alternative cobenefits from reductions in PM_2.5_, we estimated that the overall benefits for a 0.065 ppm ozone standard range from $10 billion to $21.9 billion (Table 3), in contrast to EPA’s $19 to $38 billion.

### 4.2. Benefits Approach #2

It is crucial to incorporate some measure of uncertainty into the benefits analysis, and there are multiple possible approaches that can be taken. Our second alternative benefits approach involved work by Cox (2012) [25], which discusses four important discrete assumptions that EPA makes when monetizing epidemiology study results:

(1) That there is a true statistical concentration-response association between ozone or PM_2.5_ and the health effect (i.e., that confounders are not causing a false concentration-response association).
(2) That there is a causal relationship between ozone or PM_2.5_ and the health effect (even if there is a statistical association between the two, it does not mean that the two are causally related).
(3) That there is a linear, no-threshold concentration-response relationship between ozone or PM_2.5_ and the health effect.
(4) That the concentration-response function will be the same in the future (i.e., that changes in disease prevention and medical treatments will not change the relationship between ozone or PM_2.5_ and the health effect).

Because the EPA assumed that these factors are always true, they did not calculate any variation in their benefits estimates based on any alternative interpretation. However, as established above, there is considerable uncertainty in these assumptions.

In the same paper, Cox (2012) [25] provided some suggestions for applying more realistic assumptions to benefits estimates. For example, instead of declaring that each of these assumptions is 100% correct, one could assume that there is in fact only a 50:50 chance that each is correct. This uncertainty would then be applied to the benefit estimate as detailed below. We are not assuming statistical independence between whether each assumption holds or fails. While a probability of 0.5 is being used for this example, any probability between 0 and 1 could be selected. For example, the confidences in study results discussed above could be used to choose these probabilities.

e.g., baseline monetary benefit from reducing mortality (using Smith et al. (2009) [16]) at an ozone standard of 0.065 ppm = $6.4 billion.

Uncertainty Analysis:

$6.4 billion × 0.5 (probability that true association exists)
× 0.5 (probability that association is causal)
× 0.5 (probability that there is no threshold in the response or that ambient concentrations are above the threshold)
× 0.5 (probability that the relationship is unchanged in the future)
Total = $400 million

Following Cox (2012) we estimated a second alternative measure of benefits (Supplemental Table S3). This alternative reveals a total net benefit of $1.2 to $2.5 billion, which is less than one-tenth of the $19 to $38 billion estimated by the EPA. These example calculations show how uncertainty can be considered when estimating health benefits. For a final comparison, Table 3 reports the monetized benefits from ozone reduction as estimated by the EPA, as well as our two alternative measures.

## 5. Costs of Ozone Abatement

In the draft and final RIAs released in 2014 and 2015, the EPA sought to lower ground level ozone concentrations through the mitigation of the ozone precursor NO_x_ [9,10]. Without new or significantly updated local abatement programs, the EPA predicted that 13 counties would not meet a 0.070 ppm ozone standard in 2025, and 67 counties would not meet the proposed ozone standard of 0.065 ppm by 2025 [9]. In their draft RIA, the EPA projected that without further tightening of the ozone standard, the baseline level of NO_x_ emissions would fall to 5.75 million tons annually in 2025. In order to meet the 0.065 ppm standard, the EPA estimated that annual emissions of NO_x_ must fall by 1.88 million tons to 3.87 million tons [9]. Excluding abatement methods costing more than $14,000/ton [9], the EPA identified methods to mitigate 1.12 million tons of NO_x_ each year, or about 59% of the abatement necessary to meet the proposed 0.065 ppm standard [9] (pp. 7–29). The remaining 0.75 million tons (or 41%) had to come from unknown methods (called unidentified methods in the final RIA [10]), most of which must take place in or near nonattainment areas.

To estimate the costs of these unidentified NO_x_ emission reduction methods, the EPA used the price of emission reduction credits (ERCs), or offset prices, faced by those seeking to increase NO_x_ emissions in the nonattainment areas [9] (pp. 7–22). The EPA used offset prices for four regions between 2000 and 2013: San Joaquin Valley and the South Coast in California, Houston, Texas, and the New York City region. The EPA converted these offset prices to annualized values to estimate the cost of unidentified methods to be $15,000/ton (2011$). However, the EPA’s method has two problematic assumptions: (1) that the mitigation costs do not increase as abatement increases, and (2) that offset prices serve as a reasonable proxy for cost.

### 5.1. Hybrid Cost Approach versus Average Cost Approach

To estimate the abatement cost of future unidentified controls, the EPA traditionally uses what it calls a “fixed cost methodology.” What economists might call an average cost estimate, the “fixed cost methodology” assumes each unit of abatement costs the same.

The EPA’s 2008 Ozone NAAQS RIA introduced an alternative “hybrid approach” that estimates an average and marginal cost curve for unidentified abatement controls [72]. Most notably, the hybrid approach recognizes that areas needing larger emission reductions face higher costs-per-ton and that these costs per ton rise as more abatement is necessary. Unfortunately, the 2014 draft RIA [9] and the 2015 final RIA [10] failed to build on this innovative hybrid methodology. Instead, the EPA followed the guidance of their Science Advisory Board (SAB) Advisory Council that recommended the use of the “simple, transparent” fixed cost (average cost) method because it is the “most straightforward” [73] (charge question 2a). The EPA further supported the use of the fixed cost method because “the hybrid methodology assumes all emissions reductions come from the highest cost margin of the abatement supply curve which … is unlikely for much of the unobserved abatement capacity in the present and future…. When new abatement opportunities are identified in other sectors, they typically are not at the higher end of the cost curve” ([9], pp. 7–28).

Much of this is true. For most regulations, the EPA routinely imposes ever more stringent abatement technologies on a small number of heavily regulated sectors. This has, in turn, raised the cost of abatement. However, NAAQS rules are different. NAAQS rules require all regions to meet a minimum ambient air standard. Nonattainment areas must submit emissions reduction plans to the EPA. These plans likely identify and use lower cost methods available in less regulated industries. The EPA even admits as much: “For areas needing significant additional emission reductions, much pollution abatement is likely needed from sources within regulated sectors that historically have not been intensively regulated” ([9], pp. 7–28). Therefore, the unfortunate reality is that nonattainment areas have already turned to “sectors that historically have not been intensively regulated” in order to meet or attempt to meet previous NAAQS rules. If the EPA is to remain consistent throughout its analysis, they should use the hybrid methodology introduced in the 2008 Ozone NAAQS RIA to estimate the costs of unidentified controls [72].

Moreover, the EPA reported a marginal abatement cost curve (MACC) for known controls, even though much of the cost will come from unidentified controls. By generating MACCs for unidentified controls, especially for nonattainment areas, the EPA would be able to produce more realistic cost estimates.

### 5.2. Offset Prices

Using offset prices as a proxy for the costs associated with abatement from unidentified controls is highly suspect. First, these prices likely represent either the opportunity cost of emission abatement or abatement avoidance, whichever is lowest. If it reflects abatement avoidance, then these offset prices reflect the cost of moving production from a nonattainment zone to an attainment zone. However, under this new stricter standard, more regions will be classified as nonattainment areas, thus limiting abatement avoidance through relocation. Second, if sources are unable to relocate, then these offset prices likely represent known control methods—not unknown methods. Third, all the offset prices come from current nonattainment areas. To meet the current standard, further abatement is required, which will raise offset prices above current rates. Therefore, offset prices may provide some information about the cost of known controls for the current standard in attainment areas—but not for unidentified controls for a future standard in nonattainment areas.

The rate of increase in abatement costs for newly classified nonattainment areas, however, may also be underestimated by these offset prices. The offset prices used by the EPA are generated in densely populated regions that are home to a larger number and greater variety of NO_x_ sources than a typical region. In many of the newly designated nonattainment locations there will be a limited number of available abatement methods. Given the greater diversity of abatement methods available, the regions in which these offset prices are generated likely face a slower rate of increase in marginal abatement costs than areas with a limited number of abatement options. The result is that newly designated nonattainment areas will likely face a higher rate of cost increase than the areas from which these offset prices are generated.

## 6. Alternative Measures of Costs

### 6.1. Harrison et al. Analyses

Given the concerns over the assumptions used by the EPA to estimate the costs of unidentified NO_x_ abatement methods, other groups have attempted to identify mitigation sources and their costs for attaining alternative ozone standards [74,75,76,77]. In 2014 a team of researchers at NERA Economic Consultants, Harrison et al. (2014) [74], published a study on behalf of the National Association of Manufacturers. Using the EPA’s 2008–2010 ozone rule proposals as the basis for a total mitigation requirement of 3.9 million tons of NO_x_, Harrison et al. (2014) attempted to identify and estimate the cost of all control measures necessary to meet a 0.060 ppm standard. Harrison et al. (2014) replaced the EPA’s unidentified NO_x_ abatement methods with identified retirements of coal fire power plants and older, less efficient passenger vehicles. Harrison et al. (2014) used EPA’s (2014) 2011, 2018, and 2025 Emissions State Sector Comparison data [78] to construct estimates that assume a slightly earlier compliance date of 2018 relative to EPA’s compliance date of 2020 in the 2008–2010 proposals. For power plant retirements, Harrison et al. (2014) found that NO_x_ mitigation “will cost an average of approximately $31,000/ton, but with costs ranging up to about $180,000/ton among the states” ([74], p. 16). Through the early replacement of the highest-NO_x_ emitting vehicles, Harrison et al. (2014) estimated that the marginal abatement cost of the final ton necessary to reach a 10% reduction is $100,000/ton and “about $500,000/ton to achieve about a 50% reduction” ([74], p. 16). By re-estimating the EPA’s known costs and replacing the EPA’s unknown costs with estimated costs on a state-by-state basis, Harrison et al. (2014) estimated that the reduction in NO_x_ emissions could reduce the annual U.S. gross domestic product by as much $270 million ([74], Figure 11) for a 0.060 ppm standard.

Shortly after Harrison et al. (2014) published their alternative cost measure to meet a 0.060 ppm standard, the EPA released its 2014 ozone RIA, noting a lower baseline level of NO_x_ emissions and generally focusing on a 0.065 ppm standard [9]. Both changes lowered the necessary NO_x_ mitigation to meet the 0.065 ppm standard from 3.9 million tons of abatement, to only 1.88 million tons. The EPA estimated that 1.12 million tons could be abated through known sources and 0.75 million tons from unknown sources [9]. Instead of an annualized cost of $32 to $44 billion (2006$) in 2020 to meet a 0.065 ppm standard as reported in the EPA 2008 RIA [72], the EPA estimated an annualized cost of $15 billion (2011$) to meet a 0.065 ppm standard in 2025 [9]. The EPA no longer included its “hybrid methodology” to estimate unknown costs, which averaged $36,000/ton (or $39,000/ton including California) in the 2008 proposal, but instead used an average cost of $15,000/ton for all non-California NO_x_ reductions.

After the EPA released their ozone proposed rule in 2014 [14], Harrison et al. (2015) updated their state-specific cost curve analysis to estimate the cost of meeting a 0.065 ppm standard [75]. Harrison et al. (2015) assumed that 1 million tons of NO_x_ needed to be abated through unknown methods. They created state-specific marginal cost of abatement curves by first looking at low-cost known controls that range from $0 to about $20,000 per ton. The second segment of the cost curves reflected the costs of closing coal fire power plants. Harrison et al. (2015) found that “the majority of the NO_x_ emission reductions associated with the EGU closures cost an average of about $16,000 per ton, and range well above $30,000 per ton in some states” ([75], p. 9 footnote 10). The third segment of these cost curves was based on removing pre-2008 model year light-duty vehicles still on the road in 2022. Harrison et al. (2015) estimated that those older cars would account for “about 40% of projected light-duty vehicle emissions in 2022” ([75], p. 8 footnote 9). To meet the 0.065 ppm standard, Harrison et al. (2015) found a lower annual mitigation cost estimate of $155 billion (2011$) relative to Harrison et al.’s (2014) estimate for the 0.060 ppm standard [74]. However, Harrison et al.’s (2015) cost estimate was still 10 times higher than the EPA’s estimate of $15 billion (2011 $) [9]. The Harrison et al. (2015) report does not explicitly state the annual compliance costs of the rule but does include a figure (S-5) that depicts the range of annual compliance costs. Unfortunately, the figure does not include compliance costs associated with the electric power sector which Harrison et al. (2015) model separately. The $155 billion compliance cost estimate was presented by NERA Economic Consulting in 2015 [79] and is used in the rest of this analysis.

One additional difference between Harrison et al. (2015) and the EPA’s 2014 ozone RIA is that Harrison et al. (2015) assumed that NO_x_ abatement in a particular state would lower ozone levels only in that particular state, whereas the EPA assumed that the reduction of NO_x_ at one location would lower the ozone levels at that location, throughout the state, and throughout the region. While Harrison et al. (2015) estimated mitigation of NO_x_ at the state level, the EPA [9] used five large regions covering the lower 48 states to model how NO_x_ reductions in one region lowered ozone levels across all five regions. Although this allows the EPA to spread the costs across a larger region instead of individual states, thus granting arbitrage opportunities in terms of emission abatement costs, it also provides less assurance that the level of ozone at a particular location will actually meet the new standard.

### 6.2. Fisher et al. (2015) Analysis

In September of 2015, Fisher et al. (2015) [76] and Krupnick et al. (2015) [77] published studies estimating the annualized costs of a 0.065 ppm ozone standard. Fisher et al. (2015) analyzed the EPA’s proposal as well as Harrison et al.’s cost estimates [74,75] on behalf of EarthJustice, a nonprofit environmental law organization. In their analysis, Fisher et al. (2015) points out that Harrison et al. (2015) may have double counted coal unit retirement and retrofit costs. However, Fisher et al. (2015) agrees with Harrison et al.’s (2015) assumption that economic coal plant retirements should be included in the cost estimates and that the marginal costs of NO_x_ abatement are increasing and not constant as assumed by the EPA [9]. Therefore, Fisher et al. (2015) attempted to re-estimate a national version of Harrison et al.’s (2015) state-specific cost estimates. Fisher et al. (2015) started with the EPA’s [9] estimated Base Case of 6.2 million tons of NO_x_ emissions in 2025. They then reconstructed the marginal cost of emission reductions necessary to reach a 0.065 ppm standard. Using Harrison et al. (2015), Fisher et al. (2015) constructed an unknown cost curve such that the bottom was $30,000/ton at 1.6 million tons of abatement and the top was $235,000/ton at 3.48 million tons of abatement. Their reconstructed Harrison et al. (2015) cost estimate totaled $84.5 billion, or about half of the $155 billion reported in Harrison et al. (2015).

Fisher et al. (2015) also disagreed with a number of the assumptions made by Harrison et al. (2015) (details in Appendix B). Therefore, Fisher et al. (2015) constructed their own national cost estimates. They also started with the EPA’s estimated Base Case of 6.29 million tons of NO_x_ emissions in 2025 [9] and then lowered emissions in order to meet the 0.065 ppm standard. The cost methodology used by Fisher et al. (2015) ([76], Figure 6) resulted in a marginal cost curve for unknown controls that started at $14,000 per ton, the highest cost for known controls, and arrived at $40,050 per ton for the final 660,000th ton from unknown sources. Combined with the reduction from known sources, this resulted in a total abatement of 1,942,068 tons of NO_x_. Including these adjustments to both the unknown and unknown controls, Fisher et al. (2015) found that “known controls identified by EPA and additional SCR control and retirement costs … amount to $3.7 billion (annualized). The additional cost of unknown controls … would be $17.9 billion (annualized)” ([76], p. 32). In total, “the cost of the 65 ppb ozone standard should be $21.6 billion (annualized), far below NERA’s erroneous $155 billion estimate” ([76], p. 32). Compared to the EPA’s cost estimate of $15 million [9], however, Fisher et al.’s (2015) estimate is over forty percent larger.

### 6.3. Krupnick et al. (2015) Analysis

In the fall of 2015, a team of scholars at Resources for the Future also generated an abatement cost estimate for unidentified methods for NO_x_ reduction [77]. Krupnick et al. (2015) share Fisher et al.’s (2015) view that Harrison et al. (2014, 2015) failed to properly account for the Clean Power Plan Rule’s future NO_x_ reductions, thereby increasing the abatement and corresponding costs associated with the ozone rule. Moreover, they noted that several other assumptions by Harrison et al. (2015), including using a different compliance year and reclassifying 200,000 tons identified by the EPA as unidentified, required 35% more abatement to meet the 0.065 ppm standard than does the EPA. Therefore, Krupnick et al. (2015) modified a number of assumptions from Harrison et al. (2015) to generate an alternative measure of unidentified costs (details in Appendix B). Ultimately, Krupnick et al. (2015) estimated that 420,000 tons of NO_x_ could be abated through the existing cap and trade program at an average cost of $7100 per ton. This estimate includes the Clean Power Plan but does not include mandatory selective catalytic reduction at all plants. For the remaining 530,000 tons of unidentified NO_x_ abatement, Krupnick et al. (2015) constructed a modified vehicle retirement marginal cost curve with endpoints of $7,100 per ton and $94,000 per ton that resulted in an estimate of unknown control costs that was just $1 billion higher than the EPA’s estimate.

### 6.4. Our Preferred Cost Estimate

To construct our preferred cost estimates we used the EPA’s [9] cost estimates for known mitigation methods (details of our cost estimate can be found in Appendix B). We then modified Harrison et al.’s (2015) assumptions to construct our preferred marginal abatement cost curve for identified abatement methods. To estimate the NO_x_ abatement required from unknown/unidentified sources in order to meet the 0.065 ppm standard, we used the EPA’s Draft RIA estimated Base Case of 6.29 million tons of NO_x_ emissions in 2025 [9] and then lowered emissions by:

1. Following Fisher et al.’s (2015) capture reduction assumption of 0.18 million tons from the final Clean Power Plan, instead of the EPA’s capture reductions of 0.31 million tons;
2. Following EPA’s method [9] to capture reductions from Texas and California to meet the current 0.075 ppm standard (0.24 million tons);
3. Following EPA’s method [9] to capture reductions from all EPA known controls except at electric generating units (0.92 million tons);
4. Following EPA’s method [9] to capture reductions for SCR technology (0.20 million tons);
5. Not assuming all coal-fire EGUs that emit above 0.17 tons NO_x_/MMBtu would require advanced controls or retire;
6. Not assuming all units below 250 MW or 50% capacity factor would retire economically, at the same cost as the retrofit, to be replaced with a controlled natural gas combined cycle plant;
7. Assuming that the remaining unidentified controls are met using a cost curve that:
  7.1 Starts at $15,000/ton, which is the EPA’s estimate of the average cost per ton for NO_x_ offsets. This estimate is similar to Fisher et al.’s (2015) $14,000/ton, higher than Krupnick et al.’s (2015) $7,100/ton, and lower than Harrison et al.’s (2015) $30,000/ton; and
  7.2 Follows Krupnick et al. (2015) by using a $94,000/ton marginal cost to mitigate the 3,480,000th unit of NO_x_ through a vehicle retirement program.

Supplemental Table S4 shows our mitigation assumptions along with those made by the EPA [9], Harrison et al. (2015) [75], Fisher et al. (2015) [76], and Krupnick et al. (2015) [77].

Using our marginal abatement cost curve for unidentified sources we estimated a cost for unknown controls of $29.5 billion. Adding this value to the EPA’s estimate of mitigation cost from known sources of $3.7 billion, we estimated that the total cost of NO_x_ mitigation for 2025 would be $33 billion. Table 4 reports all the cost estimates of attaining the 0.065 ppm standard. Our preferred cost measure lies below Harrison et al.’s (2015) $155 billion, but is higher than the EPA’s draft estimate of $15 billion [9], Krupnick et al.’s (2015) value of $16 billion, and Fisher et al.’s (2015) $22 billion.

## 7. Net Benefits

Using our range of benefits and the five cost estimates to attain the 0.065 ppm standard [9,75,76,77], Table 5 shows that the estimated net benefits range from −$139 to $23 billion (2011$). Of the net benefits estimated when using the EPA’s preferred measure of benefits at a 7% discount rate, only two combinations result in a range that include only positive net benefits: the EPA’s costs and Krupnick et al.’s (2015) costs. The remaining three cost estimates result in possible negative net benefits. Using our preferred measure of cost results in a range of −$14 to $4.8 billion.

Using our preferred measure of benefits reveals that all five scenarios include a possible net loss to society. The range of net benefits includes positive values for the costs estimated by the EPA, Fisher et al. (2015), and Krupnick et al. (2015). The highest estimated net benefits of $23 billion take place when using the EPA’s costs. However, the EPA’s estimated net benefits may also be as low as −$4 billion when combined with our preferred estimate of benefits. Our preferred cost estimate combined with our preferred benefits estimate results in a loss to society of between $13 and $22 billion, suggesting that society would be better off without this ozone rule in place. Following Cox’s benefit methodology [25] reveals negative net benefits across the board. The smallest loss is $13 billion. Combining our preferred cost measure with this second alternative measure of benefits generates a net benefits range of −$31 to −$32 billion.

## 8. Final Ozone Rule Update

While crafting our preferred measure of benefits and costs, the EPA issued their final rule on ozone, setting the new standard at a level of 0.070 ppm [15]. The final rule’s RIA reduced the estimated tons of NO_x_ mitigation required to meet the 0.070 ppm and the alternate 0.065 ppm ozone standards [10]. However, the EPA increased the estimated number of tons that would be mitigated via unidentified methods. In addition, they adjusted their cost estimates and performed alternative analyses to support their estimated costs of unidentified abatement methods.

In order to meet a 0.065 ppm standard, the EPA estimated that 1,380,000 tons of NO_x_ would need to be mitigated [10]. Using their Control Strategy Tool (CoST), the EPA identified potential NO_x_ abatement methods and estimated the engineering and operating costs for identified mitigation controls. The EPA used a cut-off of $19,000/ton because “controls above this value are not likely to be cost-effective”, and identified mitigation methods for 560,000 tons of NO_x_ at a cost of $2.6 billion ([10], pp. 4–6). To estimate the cost from mitigating the remaining 860,000 tons of NO_x_ through unidentified methods, the EPA cited offset prices used in the draft RIA, noted that 97% of the emissions reductions in CoST are available at a cost of less than $15,000/ton, and performed several alternative estimation strategies to support the use of an average abatement cost of $15,000/ton of NO_x_. The results from the EPA’s regression estimation and random draw strategies from Table 4A-10 in the final ozone RIA [10] are shown in Supplemental Table S5 [10]. Our Appendix B also notes several weaknesses in the methodology used to support the EPA’s preferred abatement cost estimate of $15,000 per ton for unidentified methods [10]. This resulted in $12.6 billion in unidentified costs. Adding these $12.6 billion in costs to the $2.6 billion in costs from identified methods resulted in an overall cost of $16 billion (the EPA rounds to two significant digits before adding the two types of costs [10]).

To construct our updated preferred cost estimates for unidentified controls, we first used the EPA’s estimated tons of unidentified NO_x_ abatement required for each standard: 97,000 tons to meet a 0.070 ppm standard and 960,000 tons to meet a 0.065 ppm standard [10]. Given the different methods used by the EPA to construct their cost estimate ($15,000/ton for unidentified methods and a maximum of $19,000/ton for identified methods), we used two different minimum points, $15,000/ton and $19,000/ton, when constructing our marginal costs curves. For the 0.065 ppm standard, we used the same maximum value of $94,000/ton (Section 6.4) to estimate unidentified costs.

However, to provide a preferred unidentified abatement cost estimate for the 0.070 ppm standard, we had to use a different maximum point on our marginal cost curve to account for the fact that a) 663 counties play a role in reducing NOx levels in the 14 counties that do not meet the 0.070 standard, whereas 1170 counties play a role in reducing levels to 0.065 ppm in 50 counties; and b) the 14 counties that require further, unidentified abatement methods to meet the 0.070 ppm standard have likely exhausted many of the lower cost methods of abatement available to areas that meet the 0.070 ppm standard. Therefore, we used a maximum value of $140,700/ton, or the average cost for the top 3% of abatement methods included in the EPA’s CoST data, to recognize that these locations likely face much higher marginal abatement costs. We acknowledge that, in some cases, the costs reported in the CoST data are for NO_x_ controls that were developed to abate other pollutants, such as SO_2_, and are therefore costlier than NO_x_-specific controls. However, many counties will likely have exhausted many of these known NO_x_ and unknown NO_x_-specific methods prior to meeting the 0.070 ppm standard. Supplemental Table S6 reports our preferred estimates of unidentified control costs. For the 0.070 ppm standard, we estimated that unidentified abatement costs would range from $0.8 to $0.9 billion. For the 0.065 ppm standard, unidentified NO_x_ abatement costs ranged from $28 billion to $31 billion in 2025.

To compare our methodology with the EPA’s updated measure of total non-California costs, we started with the EPA’s measure of identified costs and then used the marginal abatement cost curves above to re-estimate unidentified costs. We followed the final rule’s assumption that 860,000 tons of NO_x_ must be mitigated via unidentified methods to meet the 0.065 ppm standard, and 46,000 tons of unidentified NO_x_ abatement is required to meet the 0.070 ppm standard. Our overall cost estimates are shown in Supplemental Table S6. For the 0.065 ppm standard, we estimated that total costs would range from $31 to $34 billion (similar to our cost estimate of $33 billion for the proposed rule). For the 0.070 ppm standard, we estimated that costs would range from $1.5 to $1.6 billion.

The EPA’s final ozone rule RIA also revised the expected benefits of a 0.065 ppm standard downward by about 20%. In the proposed rule, the EPA estimated that benefits would range from $19 to $38 billion [9]. The final rule RIA estimated that benefits would range between $15 and $30 billion [10]. Supplemental Table S7 shows the EPA’s revised 0.065 ppm standard benefits estimates along with our two alternative measures of benefits. Our preferred estimate, Alternative 1, reveals a benefit range of $8 to $14 billion. Using Cox (2012) as a guide [25], our Alternative 2 reveals a benefit range of $1 to $2 billion. Table 6 repeats the exercise for the 0.070 ppm standard, showing an estimate of $1.2 to $3.3 billion for Alternative 1, and $0.2 to $0.4 billion for Alternative 2.

Supplemental Table S8 combines the revised estimates of costs and benefits for the 0.065 ppm standard to estimate the net benefits that would be generated. This analysis reveals that all combinations of costs and benefits include the possibility that a 0.065 ppm standard will result in a net loss to society. The EPA estimates that the net benefits will be between −$1 and $14 billion. Combining the EPA’s measure of benefits with a mitigation cost curve starting at $15,000/ton results in a net benefits range of −$16 to −$1 billion. Using $19,000/ton as a starting point for our abatement cost curve generates a net benefits range of −$19 to −$3.7 billion. Using our preferred measure of benefits with the EPA’s costs estimates generates a net benefits range of −$8 to $2 billion. All remaining combinations of benefits and costs result in losses for society.

Ultimately, the EPA set the new ozone standard at a level of 0.070 ppm [15]. Table 7 replicates Supplemental Table S8 but uses the final 0.070 ppm standard instead of the more stringent 0.065 ppm level. The EPA estimated that the 0.070 ppm standard would provide $1.5 to $4.5 billion in net benefits. Using our preferred measure of benefits, Alternative 1, and our two cost estimates, we founnd that the net benefits from a 0.070 ppm standard would range from −$0.2 to $1.9 billion. That is, even this less stringent standard may impose a net cost on society. Using our Alternative 2 measure of benefits results in only net costs.

## 9. Conclusions

In this paper we discussed some considerations that can be used to assess the epidemiology studies that are the basis of the EPA’s 2015 ozone NAAQS benefits estimates. These considerations include bias, confounding, evidence that the association is due to chance, integration of evidence, and applicability to future population risk. We proposed two alternative methods for calculating benefits that consider study confidence, with the result that total benefits for a 0.070 ppm standard range from $0.2 to $3.3 billion, compared to EPA’s $2.9 to $5.9 billion (based on the Final RIA [10]).

We also reviewed the EPA’s costs of an ozone standard of 0.065 ppm, as well as the cost estimates generated by three other groups [75,76,77]. We generated our own costs estimates, which differed from the EPA’s because we used a marginal cost curve (instead of a flat average cost) to estimate the costs of unidentified controls. Based on the draft RIA published in 2014 [9], we estimated a cost for attaining a 0.065 ppm ozone standard of $33 billion, which contrasts with the EPA’s estimate of $15 billion. Other estimates for attaining the ozone standard of 0.065 ppm (based on the draft RIA) ranged from $16 to $155 billion [75,76,77]. Updating our calculations based on the final RIA [10] we estimated an alternative net benefit of between -$0.3 and $1.8 billion for a 0.070 ppm standard (2011$, 7% discount rate) and between −$23 and −$17 billion for a 0.065 ppm standard. This work demonstrates that alternative reasonable assumptions can generate very difference cost and benefits estimates that may impact how policy makers view the outcomes of a major rule.

## Figures and Tables

**Table 1:** Confidence in Short-Term Ozone Mortality Studies and Long-Term PM_2.5_ Mortality Studies Used by the EPA to Derive Benefits from Decreasing the Ozone NAAQS.

Considerations	Confidence			
	Ozone Studies		PM_2.5_ Studies	
	Smith et al. 2009 [16]	Zanobetti & Schwartz 2008 [26]	Krewski et al. 2009 [57]	Lepeule et al. 2012 [58]
Exposure error	Medium	Low	Low	Low
Confounding	Medium	Low	Medium	Medium
Regional Heterogeneity	Medium	Medium	Low	Low
Consistency	Medium	Medium	Medium	Medium
Thresholds	Medium	Low	Low	Medium
Coherence	Low	Low	Low	Low
Recent Data	Low	Low	Low	Medium
**Overall**	**Medium**	**Low**	**Low**	**Medium**

**Table 2:** Example of Probably Weights Applied to Seven Confidence Areas of Smith et al. (2009), Based on Confidence Ratings.

Considerations	Confidence	Probability *	Weight	Total
	Smith et al. (2009)			
Exposure error	Medium	0.75 (0.6–0.9)	0.1429	0.1072 (0.0857–0.1286)
Confounding	Low	0.4 (0.3–0.5)	0.1429	0.0572 (0.0429–0.0715)
Regional Heterogeneity	Medium	0.75 (0.6–0.9)	0.1429	0.1072 (0.0857–0.1286)
Consistency	Medium	0.75 (0.6–0.9)	0.1429	0.1072 (0.0857–0.1286)
Thresholds	Medium	0.75 (0.6–0.9)	0.1429	0.1072 (0.0857–0.1286)
Biological Plausibility	Low	0.4 (0.3–0.5)	0.1429	0.0572 (0.0429–0.0715)
Recent Data	Low	0.4 (0.3–0.5)	0.1429	0.0572 (0.0429–0.0715)
**Overall**	**Medium**			**0.6004 (0.4715–0.7289)**

* The low corresponding value weights range from 0.3 to 0.9 while the high corresponding weights range from 0.5 to 1.1.

**Table 3:** Estimated Monetized Ozone and PM_2.5_ Benefits for a 0.065 ppm standard: EPA, Alternative 1, and Alternative 2 (billions of 2011$) ^a^.

	EPA RIA 2014 [9]	Alternative 1	Alternative 2
Ozone-only benefits ^b^	$6.4 to $11	$3.8 to $5.5	$0.4 to $0.7
PM_2.5_ cobenefits ^c^	$12 to $28	$7.2 to $14	$0.8 to $1.8
**Total Benefits**	**$19 to $38**	**$11 to $20**	**$1.2 to $2.5**

^a^ Rounded to two significant figures. The reduction in premature fatalities each year accounts for over 98% of total monetized benefits in this analysis. Not all possible benefits are quantified and monetized in this analysis; ^b^ range reflects Smith et al. (2009) [16] and Zanobetti & Schwartz (2008) [26]. Ozone-only benefits reflect short-term exposure impacts and as such are assumed to occur in the same year as ambient ozone reductions. Consequently, social discounting is not applied to the benefits for this category; ^c^ range reflects Krewski et al. (2009) [57] and Lepeule et al. (2012) [58]; 7% discount rate. Mortality risk valuation for PM_2.5_ assumes discounting over the SAB-recommended 20-year segmented lag structure.

**Table 4:** Monetized Cost of Tons of NO_x_ Mitigation (in billions 2011 $) (compliance year in parentheses).

	EPA Draft RIA 2014 (2025) [9]	Harrison et al. 2015 (2022) w/CA ^b^	Fisher et al. (2025)	Krupnick et al. (2025) ^a^	Lange et al. (2025)
**Identified Costs**	$3.8		$3.7	$3.8	$3.7
**Unidentified Costs**	$11.0		$17.9	$12.0	$29.5
**Total Cost of Emissions Reduction**	**$15**	**$155**	**$22**	**$16**	**$33**

^a^ Krupnick et al. (2015) did not report their estimate of Identified Costs—EPA’s estimate used in its place; ^b^ Leveraged on an average annualized basis from 2018 to 2040.

**Table 5:** Net Benefits Based on EPA’s Estimate of Monetized Ozone and PM_2.5_ Net Benefits for Proposed Annual Ozone Standard of 0.065 ppm 2025 Scenario [9] (nationwide benefits of attaining each alternative standard everywhere in the U.S. except California and except as noted; compliance year in parentheses)—Full Attainment (billions of 2011$) ^a^.

Total Benefits ^a^		Costs		Net Benefits ^c^
Source		Source (Compliance Year)	Total Costs	
EPA Draft RIA (2014)	$19 to $38	EPA RIA 2014 (2025) [9]	$15	$4.0 to $23
		Harrison et al. 2015 (2022)	$155	($136) to ($117)
		Fisher et al. 2015 (2025)	$22	($3.0) to $16
		Krupnick et al. 2015 (2025) ^b^	$16	$3.0 to $22
		Lange et al. 2018 (2025)	$33	($14) to $4.8
Lange et al. Alternative 1	$11 to $20	EPA RIA 2014 (2025) [9]	$15	($4.0) to $5.0
		Harrison et al. 2015 (2022)	$155	($144) to ($135)
		Fisher et al. 2015 (2025)	$22	($11) to ($2.0)
		Krupnick et al. 2015 (2025) ^b^	$16	($5.0) to $4.0
		Lange et al. 2018 (2025)	$33	($22) to ($13)
Lange et al. Alternative 2	$1.2 to $2.4	EPA RIA 2014 (2025) [9]	$15	($14) to ($13)
		Harrison et al. 2015 (2022)	$155	($154) to ($153)
		Fisher et al. 2015 (2025)	$22	($21) to ($20)
		Krupnick et al. 2015 (2025) ^b^	$16	($15) to ($14)
		Lange et al. 2018 (2025)	$33	($32) to ($31)

^a^ 7% discount rate; ^b^ Krupnick did not report their estimate of Identified Costs—EPA’s estimate used in its place; ^c^ parentheses indicate negative numbers.

**Table 6:** Estimated Monetized Ozone and PM_2.5_ Benefits for Revised and Alternative Annual Ozone Standards of 0.070 ppm using the EPA Final Ozone RIA [10] Table 6–24 as the basis (in billions of dollars) ^a^.

	EPA RIA (2015) [10]	Lange et al. Alternative 1	Lange et al. Alternative 2
Ozone-only benefits ^b^	$1.0 to $1.7	$0.6 to $0.8	$0.1 to $0.1
PM_2.5_ Cobenefits ^c^	$1.9 to $4.2	$0.6 to $_2.5_	$0.1 to $0.3
**Total Benefits**	**$2.9 to $5.9**	**$1.2 to $3.3**	**$0.2 to $0.4**
**Identified Control Strategies Only**			
Ozone-only benefits ^b^	$0.9 to $1.4	$0.5 to $0.7	$0.1 to $0.1
PM_2.5_ Cobenefits ^c^	$1.6 to $3.5	$0.8 to $2.1	$0.1 to $0.2
**Total Benefits**	**$2.4 to $4.9**	**$1.3 to $2.8**	**$0.2 to $0.3**

^a^ Rounded to two significant figures. The reduction in premature fatalities each year accounts for over 98% of total monetized benefits in this analysis. Mortality risk valuation for PM_2.5_ assumes discounting over the SAB-recommended 20-year segmented lag structure. These estimates reflect the economic value of avoided morbidities and premature deaths using risk coefficients from the studies noted; ^b^ Range reflects Smith et al. (2009) and Zanobetti & Schwartz (2008). Ozone-only benefits reflect short-term exposure impacts and as such are assumed to occur in the same year as ambient ozone reductions. Consequently, social discounting is not applied to the benefits for this category; ^c^ range reflects Krewski et al. (2009) and Lepeule et al. (2012). 7% discount rate.

**Table 7:** Estimate of Monetized Ozone and PM_2.5_ Net Benefits for Annual Ozone Standard of 0.070 ppm 2025 Scenario, using EPA (2015) [10] as the basis (nationwide benefits of attaining each alternative standard everywhere in the U.S. except California and except as noted)—Full Attainment (billions of 2011$) ^a^.

Total Benefits ^a^		Costs			Net Benefits ^b^
Source		Source	Minimum Cost of Initial Unidentified Method (per ton)	Total Costs	
EPA Final RIA (2015)	$2.9 to $5.9	EPA (2015) RIA [10]	$15,000	$1.4	$1.5 to $4.5
		Lange et al. using EPA (2015) RIA [10]	$15,000	$1.5	$1.4 to $4.4
		Lange et al. using EPA (2015) RIA [10]	$19,000	$1.6	$1.3 to $4.3
Lange et al. Alternative 1	$1.2 to $3.3	EPA (2015) RIA [10]	$15,000	$1.4	($0.2) to $1.9
		Lange et al. using EPA (2015) RIA [10]	$15,000	$1.5	($0.3) to $1.8
		Lange et al. using EPA (2015) RIA [10]	$19,000	$1.6	($0.4) to $1.7
Lange et al. Alternative 2	$0.2 to $0.4	EPA (2015) RIA [10]	$15,000	$1.4	($1.2) to ($1.0)
		Lange et al. using EPA (2015) RIA [10]	$15,000	$1.5	($1.3) to ($1.1)
		Lange et al. using EPA (2015) RIA [10]	$19,000	$1.6	($1.4) to ($1.2)

^a^ 7% discount rate; ^b^ parentheses indicate negative numbers.

## Supplementary Materials

The following are available online at http://www.mdpi.com/1660-4601/15/8/1586/s1,, Supplemental Table S1: Total Annual Costs and Benefits for the U.S. in 2025, and for California post-2025 (billions of 2011$, 7% discount rate). Numbers taken from Tables ES-6 and ES-10 in the Draft Ozone RIA and Tables ES-5 and ES-9 in the Final Ozone RIA. Supplemental Table S2: EPA Estimation and Alternative 1 Re-estimation of Ozone Benefits and PM_2.5_ Cobenefits for 2025 (non-California estimates, millions of 2011$); (EPA numbers from EPA Draft Ozone RIA Table 5–20 for ozone and Table 5-22 for PM_2.5_). Supplemental Table S3: Alternative Benefits Calculations Assuming Uncertainty in Health Studies. Supplemental Table S4: Millions of tons of NO_x_ Emission Reductions from EPA’s Draft RIA, Harrison, et al. (2015), Fisher, et al. (2015), Krupnick et al. (2015), and Lange et al. (2018) (Compliance Year in Parentheses). Supplemental Table S5: Unidentified NO_x_ Control Costs by Alternative Standard using Alternative Methods (Based on Table 4A-10 from EPA Final Ozone RIA 2015). Supplemental Table S6: Monetized Costs of Tons of NO_x_ Emissions using EPA Final Ozone RIA 2015 (in billions 2011$). Supplemental Table S7: Estimated Monetized Ozone and PM_2.5_ Benefits for Revised and Alternative Annual Ozone Standards of 0.065 ppm using EPA 2015 Final Ozone RIA Table 6–24 as the basis (in billions of dollars). Supplemental Table S8: Estimate of Monetized Ozone and PM_2.5_ Net Benefits for Proposed Annual Ozone Standard of 0.065 ppm 2025 Scenario, using EPA 2015 Final Ozone RIA as the basis (nationwide benefits of attaining each alternative standard everywhere in the U.S. except California and except as noted)—Full Attainment (billions of 2011$).

## Appendix A. Confidence Assessment in Papers Used to Inform the EPA’s Draft 2014 RIA [9] or Final 2015 RIA [10] Ozone and Particulate Matter Mortality Estimates

**Ozone Short-Term Mortality**

**Smith et al. 2009a** [16]:

Exposure measurement error: Medium confidence: This study used stationary ambient air monitors to estimate the study population’s exposure to ozone and other pollutants. There is no discussion in the methods about considerations of personal exposure or exposure error in this papers’ exposure models. However, the inclusion of air conditioners and age of home would both affect personal exposure (air conditioners and new homes with better insulation lower intrusion of outdoor air and decrease ozone exposure).

Confounding: Medium confidence: PM_10_ and SO_2_ are considered as copollutants, and multiple models for temperature were used, but there was no assessment of residual or unmeasured confounders. It should be noted that the EPA chose to use the effect estimate that did not consider copollutant confounding, and so when applying this confidence to EPA’s benefit’s estimates, we used a low confidence.

Regional Heterogeneity: Medium confidence: Extensive analyses were done on regional heterogeneity, and show a very clear regional effect of ozone on mortality (e.g. only 7 out of 98 cities show a significant effect of 8-h ozone concentrations on mortality).

Consistency: Medium confidence: Many studies (>75% of those reported in the EPA’s ISA) have shown statistically significant effects of ozone on mortality. However, multiple studies have investigated and found evidence of publication bias thereby decreasing confidence in the consistency of the results [47,48,49].

Thresholds: Medium confidence: Thresholds were discussed by the authors and a non-linear model was fit, but the authors still modeled mortality to zero (i.e., with no threshold). In addition, this study suffers from exposure measurement error and regional heterogeneity that are known to obscure thresholds even if they exist in the data [51,53].

Coherence: Low confidence: There is no MOA for mortality at ambient ozone concentrations and it is inconsistent with other human and animal data (e.g., Figure 3). In addition, it is unlikely that people in the days before their death were outside exercising, and therefore exposed to significant amounts of ozone.

Recent Data: Low confidence: The data used was from 1987–2000, so more than half of the data was more than 20 years old.

**Zanobetti & Schwartz 2008** [26]:

Exposure measurement error: Low confidence: There is no discussion in the methods about considerations of personal exposure.

Confounding: Low confidence: Temperature was considered as a confounder in several models, but no other pollutants were considered.

Regional Heterogeneity: Medium confidence: This study includes analysis of individual cities, and a regional difference in the association between ozone and mortality was seen.

Consistency: Medium confidence: Many studies (>75% of those reported in the EPA’s ISA) have shown statistically significant effects of ozone on mortality. However, multiple studies have investigated and found evidence of publication bias [47,48,49].

Thresholds: Low confidence: There was no discussion of thresholds, and all models were linear to an ozone concentration of zero. In addition, this study suffers from exposure measurement error and regional heterogeneity that are known to obscure thresholds even if they exist in the data [51,53].

Coherence: Low confidence: There is no MOA for mortality at ambient ozone concentrations and it is inconsistent with other human and animal data. In addition, it is unlikely that people in the days before their death were outside exercising, and therefore exposed to significant amounts of ozone.

Recent Data: Low confidence: the data used is from 1989–2000, so more than half of the data was more than 20 years old.

**Particulate Matter Long-Term Mortality**

**Krewski et al. 2009** [57]:

Exposure measurement error: Low confidence: There is no discussion in the methods about considerations of personal exposure, except for consideration of air conditioning use at the population level. Air pollutant data for PM_2.5_ was only available from 1979 to 1983 and from 1999 to 2000.

Confounding: Medium confidence: The authors considered income, poverty, unemployment, education, air conditioning, income disparity, and race at the population level; and smoking, marital status, body mass index, alcohol consumption, occupation, diet, education, and occupational exposure to dust and fumes at the individual level. They did not model copollutants despite having collected data from multiple air pollutants.

Regional Heterogeneity: Low confidence: This study investigated >100 cities in the U.S., with sub-analyses on the New York City and Los Angeles areas alone. Only nationwide, New York and Los Angeles effect estimates were presented, and the effects of PM_2.5_ on all-cause mortality were heterogenous between New York and Los Angeles.

Consistency: Medium confidence: Other studies have shown an association between PM_2.5_ and mortality. However, because these studies are all subject to exposure measurement error bias (made worse because air pollutant data was only available at the beginning and end of the study), the confidence in consistency is only medium.

Thresholds: Low confidence: The analysis is silent with respect to PM_2.5_ mortality thresholds, although a non-linear (logarithmic) model of PM_2.5_ effects was considered in a sensitivity analysis. In addition, this study suffers from exposure measurement error that is known to obscure thresholds even if they exist in the data [51,53].

Coherence: Low confidence: There is no MOA for mortality at ambient concentrations of PM_2.5_, and it is inconsistent with other human and animal data.

Recent Data: Low confidence: The data used is from 1982–2000, and therefore more than half of it is more than 20 years old.

**LePeule et al. 2012** [58]:

Exposure measurement error: Low confidence: There is no discussion in the methods about considerations of personal exposure. Air pollutant data for PM_2.5_ was only available from 1979 to 1988 and from 1999 to 2009. Missing years of PM_2.5_ data was interpolated from PM_10_ and visibility data.

Confounding: Medium confidence: The study included individual confounders such as smoking, education, and body mass index, but no copollutants.

Regional Heterogeneity: Low confidence: This study was conducted in six cities, but no results are presented to address potential regional heterogeneity.

Consistency: Medium confidence: Other studies have shown an association between PM_2.5_ and mortality. However, because these studies are all subject to exposure measurement error bias (in particular because much of the air pollution data must be interpolated over the timescale of the study), the confidence in consistency is only medium.

Thresholds: Medium confidence: The authors tested for a threshold for mortality and concluded that a linear concentration response was indicated using their method. In addition, this study suffers from exposure measurement error that is known to obscure thresholds even if they exist in the data [51,53].

Coherence: Low confidence: There is no MOA for mortality at ambient concentrations of PM_2.5_, and it is inconsistent with other human and animal data.

Recent Data: Medium confidence: The data used is from 1979 to 2009, so more than half the data is more than 10 years old.

## Appendix B. Details of Cost Estimates from Fisher et al. (2015) [76], Krupnick et al. (2015) [77], Lange et al. (2018) (This Paper), and the EPA’s Final Ozone RIA [10]

### Appendix B.1. Details on Fisher et al.’s (2015) Adjustments to Harrison et al. (2015)

Fisher et al. (2015) [76] disagreed with a number of the assumptions made by Harrison et al. (2015) [75]:

- Assigning an earlier compliance deadline of 2022 that increased the amount of NO_x_ abatement that must take place in response to the ozone rule, given the EPA’s assumption that ozone levels will continue to decline without additional regulations;
- Failing to include the (now) final Clean Power Plan rule that lowered future baseline emissions by 309,000 tons and thus raised the amount of unknown (unidentified) emission controls required for compliance. A stay and proposed repeal on the final Clean Power Plan (CPP) rule means that 179,000 tons will be abated by 2025 and not the 309,000 tons estimated in the final—but currently on hold—rule; and
- The methods used to estimate identified costs through the retirement and retrofit of electric generating units (EGUs) and through the retirement of higher emitting passenger vehicles in place of the EPA’s unknown (unidentified) costs.

Therefore, Fisher et al. (2015) constructed their own national cost estimates. They started with the EPA’s Draft RIA [9] estimated Base Case of 6.29 million tons of NO_x_ emissions in 2025 and then lowered emissions in order to meet the 0.065 ppm standard by:

1. Capturing reductions for the updated base case in the final Clean Power Plan RIA (0.06 million tons);
2. Capturing reductions due to the final Clean Power Plan (0.18 million tons);
3. Capturing reductions from Texas and California meeting the current 0.075 ppm standard (0.24 million tons; same as the EPA);
4. Capturing reductions from all EPA known controls except at EGUs (0.92 million tons);
5. Assuming all coal-fire EGUs (not combined heat and power) that emit above 0.17 NO_x_/MMBtu would require advanced controls or retire;
6. Assuming that units below 250 MW or 50% capacity factor would retire economically, at the same cost as the retrofit, to be replaced with controlled natural gas combined cycle (NGCC) plants; and
7. Assuming the remaining unknown (unidentified) controls are met using a cost curve half as steep as Harrison et al.’s (2015) marginal cost of abatement, adjusted to meet the highest cost of known controls at the low end.

### Appendix B.2. Details on Krupnick et al.’s (2015) Adjustments to Harrison et al. (2015)

Krupnick et al. (2015) [77] also used Harrison et al. (2015) [75] as a starting point for their cost estimates, but made a number of adjustments. First, they used 2025 as the year of compliance to remain consistent with the EPA’s proposal. Second, they used the Resources for the Future’s Haiku electricity model [80] to assess the cost of reducing NO_x_ emissions through existing cap-and-trade programs. Krupnick et al. (2015) estimated that 420,000 tons of NO_x_ could be abated through the existing cap and trade program at an average cost of $7100 per ton (this estimate includes the Clean Power Plan but does not include mandatory selective catalytic reduction at all plants). Of these 420,000 tons, Krupnick et al. (2015) estimated that 220,000 were beyond those identified by the EPA. For the remaining 530,000 tons of unknown (unidentified) NO_x_ abatement, Krupnick et al. (2015) followed Harrison et al. (2015) and constructed a modified vehicle retirement marginal cost curve. The modification involves alternative endpoints of the marginal cost curve. Following Fisher et al. (2015), Krupnick et al. (2015) assumed that a 40% reduction in emissions, or 3.48 million tons of NO_x_ from the 6.3 million ton baseline, are possible using the vehicle retirement program after known controls account for 0.8 million tons and EGU controls account for another 0.8 million tons (1.6 million). Of the remaining 4.7 million tons to reach zero emissions, 40% is 1.88 million tons. Therefore, Krupnick et al.’s (2015) bottom end point of the unknown (unidentified) cost curve is $7100/ton at 1.6 million tons and the top of the unknown (unidentified) cost curve is $94,000/ton at 3.48 million tons. Krupnick et al. (2015):
…anchor the curve at its lowest point at $7100 per ton, which replaces Harrison et al.’s $29,000 to reflect the change from Harrison et al.’s coal retirement program to (Krupnick et al. (2015))’s trading program. Krupnick et al. (2015) anchors the curve at its highest point at $94,000, which replaces Harrison et al.’s $250,000 for a vehicle scrappage program. Krupnick et al. (2015) calculates this new maximum marginal cost by tripling the estimated costs of the Cash for Clunkers program by Li et al. (2013) [81] to reflect the lower fleet emissions in 2025. Tripling the costs accounts for the reduction in average emissions rates of the fleet over time and assumes that the program causes the retirement of vehicles that have emissions rates roughly three times the fleet-wide average (which was the case under Cash for Clunkers). ([77], p. 13)

The resulting marginal abatement cost curve defining the mitigation methods for the 750,000 tons of emission reductions unidentified by the EPA started at $7100 and remained so for the first 220,000 tons for $1.6 billion (2011$). The remaining 530,000 tons of mitigation was about $10.3 billion for a total of $12 billion or just $1.0 billion more than the EPA’s estimate of unknown (unidentified) costs.

### Appendix B.3. Details on the Lange et al. (2018; This Paper) Preferred Cost Estimate Using the EPA’s Draft Ozone RIA [9]

Generating the abatement costs from unknown (unidentified) methods involves estimating the end points of the marginal abatement cost curve. We share Krupnick et al.’s (2015) view that the national NO_x_ cap and trading market will help to reduce the cost of NO_x_ abatement by encouraging facilities with lower costs of abatement to take advantage of their low-cost abatement methods. However, a cap and trade market will only help locations meet the new ozone standard if low cost abatement methods are still available within the noncompliance area or if they can trade with areas that affect the nonattainment areas. That is, the trading can lower the total level of NO_x_ emitted, but this does not guarantee compliance at each and every location. Potential noncompliance is true for all areas and especially true for nonattainment areas that have been using cap and trade programs in the past. For example, the Northeast began a cap and trade program for boilers and power plants in 1999. Much of the Northeast is also under the jurisdiction of the multi-state Ozone Transport Commission that coordinates interstate cooperation for regional air quality planning and assists with mitigation agreements. However, the Northeast, along with California, has some of the highest costs of abatement. The Northeast is also home to many counties that do not meet the current ozone standard. Therefore, any new reduction in the Northeast will likely be costlier than in current attainment areas. Moreover, almost one third of the reductions from unidentified sources are predicted to come from the costlier Northeast.

The likelihood that the cap and trade program for boilers and power plants will result in abatement costs savings is also low for areas of the Southeast and the Midwest that began participating in the cap and trade program in 2003. For instance, according to the Georgia Department of Natural Resources, “No significant additional NO_x_ control measures are available to Georgia for the Atlanta nonattainment area. Any future reduction in NO_x_ emissions will come from federal control measures for on-road mobile sources, off-road mobile sources, locomotives, aircrafts, and shipping” ([82], p. 6). Given that known mitigation methods costing less than $15,000/ton are available for only a fraction of the NO_x_ mitigation required to meet the standard, it is unlikely that national cap and trade programs will greatly lower the mitigation costs faced by nonattainment areas that have been using such programs for over a decade. Therefore, we followed the EPA [9] and use $15,000/ton as the lowest point on our marginal abatement cost curve.

For the vehicle retirement program, we followed Krupnick et al. (2015) who cited analyses of vehicle buyback programs [81,83]. Both studies estimated that the average cost of reducing NO_x_ emissions is just over $31,000 per ton. But, as noted by Krupnick et al. (2015), the emission standards and levels of enforcement are already higher for many nonattainment areas. For example, many diesel passenger vehicles that emit higher levels of NO_x_ than gasoline vehicles were sold in only 45 states in the late 1990s and early 2000s. Therefore, a retirement program in the five states where diesel vehicles were not sold for a period of time, including California and much of the Northeast, will result in less mitigation per vehicle retired, raising the cost per ton of NO_x_ abatement. Therefore, we followed Krupnick et al. (2015) and multiplied the $31,000+/ton by a factor of three for a total value of $94,000/ton to account for the lower fleet emissions in 2025.

### Appendix B.4. Considerations for the EPA’s Final Ozone RIA [10] Cost Estimate

In the final ozone RIA the EPA noted that 97% of the emissions reductions in CoST are available at a cost of less than $15,000/ton [10]. The EPA then performed several alternative estimation strategies to support the use of an average abatement cost of $15,000/ton of NO_x_. However, many of the alternative strategies used by the EPA failed to include all the available CoST data. For instance:
“Using all observations under the cost per ton threshold for identified controls ($19,000/ton for NO_x_), a linear regression is estimated and used to predict the price of the additional unidentified controls required to attain a particular level of the standard. That is, to meet a particular level of the standard, it is assumed that all reductions that can be achieved at a cost less than the cost threshold will first be exhausted and any additional tons required can be achieved at a cost determined by the value of the regression line at those tons”. ([10], p. 4A-7)

Therefore, when attempting to estimate the costs of unidentified emission controls, the EPA dropped all the identified emission control methods with costs greater than $19,000/ton from their linear regression estimate. The EPA’s reasoning for dropping these controls is mentioned when estimating the costs of identified controls:
“Because the identified control cost curve reflects incomplete information, it is necessary to take steps to identify likely impractical control applications and to remove them from the analysis. We determined that applying an exponential trend line would produce a reasonable cost threshold for identified controls, and we used the assumption in this analysis. To determine a cost threshold for identified NO_x_ controls, we used the full dataset on NO_x_ control measures and plotted an exponential trend line through the identified control cost curve. … the curves intersect at $19,000 per ton, meaning control costs above $19,000 per ton begin increasing at more than an exponential rate. We selected $19,000 per ton as the control cost value above which we would not apply additional identified NO_x_ controls because controls above this value are not likely to be cost-effective”. ([10], pp. 4–6)

Thus, in reality, the EPA’s CoST data included known controls that are more than $19,000 per ton, but the EPA failed to include them in the identified costs, because they are “not likely to be cost-effective”. Instead, the EPA turned to unidentified emission abatement options. When estimating the costs of these unidentified emission controls with a linear regression, the EPA again dropped all the observations that were greater than $19,000/ton. The EPA supported the exclusion of abatement methods costlier than $15,000/ton by noting that the raw CoST data,
“has a median control cost of $10,400/ton and an emissions-weighted average cost of $3000/ton; 97% of the emissions reductions from these controls are available at a cost less than $15,000/ton. … Given that both the statistics on the entire data set for identified NO_x_ controls and the results of the alternative approaches for valuing unidentified controls provide costs below $15,000/ton, the decision to value unidentified NO_x_ controls at $15,000/ton is both appropriate and conservative”. ([10], pp. 4–8)

But the “statistics on the entire data set for identified NO_x_ controls” did not include all the CoST data. It only included abatement methods with costs below $19,000/ton. In addition, many of the alternative approaches also top-truncated the CoST data.

Moreover, in footnote 59 on page 4–8 the EPA stated that in the “raw data, the average control cost is $17,800/ton. This average control cost is influenced by a few very high cost control applications that we do not apply in the identified control strategy analyses.” [10]. But this is puzzling. Although it is true that 97% of the mitigation in the CoST data is less than $15,000/ton, six of the eight regions analyzed by the EPA are unable to meet the standard by using 97% of the control strategies listed in the CoST data [10]. That is, in order to meet the standard, all six nonattainment regions must utilize methods that are equal to or greater than $15,000/ton. If 97% of the mitigation methods listed in the CoST data are less than $15,000/ton, say $14,000/ton, and the average cost per ton for the entire CoST data is $17,800/ton, then the average cost for the top 3% of the CoST data set must be $140,700/ton. Recognizing that these methods are “not cost-effective”, the EPA assumed that mitigation methods yet to be identified will be 7.4 times less costly ($140,700/ton divided by $19,000/ton) than all remaining identified, but not implemented, abatement options.

To further support their average mitigation cost of $15,000/ton for unidentified methods, the EPA also performed a number of random draw strategies, repeated 1000 times to ensure consistency, to estimate the costs of unidentified methods [10]. Three random draw strategies did include the entire universe of known control methods: selected from sectors in the same proportion as the identified control strategies; selected in proportion to the available tons of reduction remaining after using identified control strategies; and selected entirely randomly. The EPA also performed random draws from the regression line beyond identified controls and from the entire regression line. The results from the regression estimation and random draw strategies from Table 4A-10 [10] are shown in Supplemental Table S4.

Ultimately, any abatement control methods remaining after known controls are applied will most likely only be available in locations that meet the standard. While using these remaining control methods may reduce the overall level of NO_x_ emitted, the areas that will need additional abatement controls to meet the standard, such as the Northeast and Great Lakes Region, will likely have exhausted all known control methods. This is implicitly noted by the EPA, when they state “while there is geographic specificity in the applicability of the controls, the simulation is currently being performed on a national scale” ([10], p. 4A-10). This is also true with our preferred measure of costs. However, our measure recognizes that 60% of the mitigation must take place at or near locations that will require additional yet-to-be identified mitigation methods–above the baseline to meet even the 0.070 ppm standard.
