# Supplementary material for: What Are the Net Benefits of Reducing the Ozone Standard to 65 ppb? An Alternative Analysis
Source: Int J Environ Res Public Health. 2018 Jul 26;15(8):1586. doi: 10.3390/ijerph15081586 (PMC6121288; doi:10.3390/ijerph15081586)
Supplement: Supplementary file 1 [file ijerph-15-01586-s001.pdf]

Supplemental Table S1: Total Annual Costs and Benefits for the U.S. in 2025, and for California post-2025 (billions of 2011$, 7% discount rate). Numbers taken from Tables ES-6 and ES-10 in the Draft Ozone RIA and Tables ES-5 and ES-9 in the Final Ozone RIA.

	**U.S. non-California Cost/Benefits in 2025**		**California Costs/Benefits post-2025**	
	**Alternative Standards**		**Alternative Standards**	
	**0.070 ppm**	**0.065 ppm**	**0.070 ppm**	**0.065 ppm**
	**Draft Ozone RIA (2014)**			
Total Costs	$3.9	$15	$0.80	$1.6
Total Health Benefits	$6.4 to $13	$19 to $38	$1.1 to $2	$2.2 to $4.1
**Net Benefits**	**$2.5 to $9.1**	**$4 to $23**	**$0.3 to $1.2**	**$0.6 to $2.5**
	**Final Ozone RIA (2015)**			
Total Costs	$1.4	$16	$0.80	$1.5
Total Health Benefits	$2.9 to $5.9	$15 to $30	$1.2 to $2.1	$2.3 to $4.2
**Net Benefits**	**$1.5 to $4.5**	**-$1.0 to $14**	**$0.4 to $1.3**	**$0.8 to $2.7**

Supplemental Table S2: EPA Estimation and Alternative 1 Re-estimation of Ozone Benefits and $PM_{2.5}$ Co-benefits for 2025 (non-California estimates, millions of 2011$); (EPA numbers from EPA Draft Ozone RIA Table 5-20 for ozone and Table 5-22 for $PM_{2.5}$).

Study	EPA Estimates		Alternative 1 Estimates	
	**0.070 ppm**	**0.065 ppm**	**0.070 ppm**	**0.065 ppm**
	**Ozone Benefits (Short-Term Mortality)**			
Smith et al. (2009)	$2,000<br>($180 to $5,800)	$6,400<br>($560 to $18,000)	$1,200<br>($110 to $3,500)	$3,800<br>($340 to $11,000)
Zanobetti & Schwartz (2008)	$3,400<br>($300 to $9,600)	$11,000<br>($950 to $30,000)	$1,700<br>($150 to $4800)	$5,500<br>($480 to $15,000)
	**$PM_{2.5}$ Benefits (Long-Term Mortality, 7% Discount rate)**			
Krewski et al. (2009)	$4,300	$12,000	$2,600	$7,200
LePeule et al. (2012)	$9,700	$28,000	$4,800	$14,000

Supplemental Table S3. Alternative Benefits Calculations Assuming Uncertainty in Health Studies.

**Health Effect & Study**	**Probability of Assumption Being True**				**Overall Probability**	**Benefit Estimate with Uncertainty (in Millions)**
	True Association?	Causal?	Linear No-Threshold?	True in the Future		
			Ozone Benefits – Short-term Mortality			
Smith et al. (2009)	0.5	0.5	0.5	0.5	0.0625	$400
Zanobetti & Schwartz (2008)	0.5	0.5	0.5	0.5	0.0625	$690
			$PM_{2.5}$ Benefits – Long-term Mortality			
Krewski et al. (2009) [a]	0.5	0.5	0.5	0.5	0.0625	$750
LePeule et al. (2012) [a]	0.5	0.5	0.5	0.5	0.0625	$1,750
		**Total Monetized Benefits**				**$1,150 to $2,500**

[a]Long-term mortality calculated using a 7% discount rate.

Supplemental Table S4: Millions of tons of NO_x Emission Reductions from EPA's Draft RIA, Harrison, et. al. (2015), Fisher, et. al (2015), Krupnick et. al. (2015), and Lange et. al. (2018) (compliance year in parentheses).

	**EPA Draft RIA (2025)**	**Harrison, et. al. (2022)**	**Fisher et. al. (2025)**	**Krupnick et. al. (2025)**	**Lange et. al. (2025)**
Base Case Emissions	6.29	6.60	6.29	6.29	6.29
Updated 2015 Base Case			(0.06)		
Reductions to CPP proposal	(0.31)			(0.31)	
Reductions to CPP final			(0.18)		(0.18)
Reductions to 75 ppb	(0.24)	(0.30)	(0.24)	(0.24)	(0.24)
Baseline Emissions	5.75	6.30	5.81	5.75	5.87
Known controls, minus EGU SCR	(0.92)	(0.80)	(0.92)	(0.92)	(0.92)
EGU SCR	(0.20)		(0.24)		(0.20)
EGU closures		(0.80)	(0.12)		
NOx Trading Program*				(0.42)	
Unknown controls	(0.75)	(1.00)	(0.66)	(0.53)	(0.88)
Compliance Emissions	3.87	3.70	3.87	3.88	3.87
**Total Reductions from Baseline**	**1.88**	**2.60**	**1.94**	**1.87**	**2.00**

* Does not exclude SCRs and other control methods

Supplemental Table S5: Unidentified NO_X Control Costs by Alternative Standard using Alternative Methods (Based on Table 4A-10 from EPA Final Ozone RIA 2015 [a]).

		Simulation Approach						Lange et. al. using EPA (2015) RIA	
**Level of Standard**	**Tons of Unidentified NO_X reductions [b,c]**	**Regression Approach**	**Sector Percentages from Applied Controls**	**Sector Percentages from Remaining Emissions**	**Random Draws from all Identified Controls**	**Random Draws from Regression Line Beyond Identified Controls**	**Random Draws from Entire Regression Line**	**Minimum Cost of Initial Unidentified Methods is $15,000/ton**	**Minimum Cost of Initial Unidentified Methods is $19,000/ton**
70 ppb	97,000	$960 (9,800)	$250 (2,500)	$310 (3,100)	$290 (3,000)	$1,400 (14,000)	$940 (9,600)	$1,770 ($1,843)	$2,148 ($2,237)
65 ppb	960,000	$14,000 (14,000)	$2,700 (2,800)	$3,000 (3,100)	$2,900 (3,000)	$14,000 (14,000)	$9,300 (9,600)	$33,763 ($35,170)	$36,623 ($38,149)

[a]All EPA values are rounded to two significant figures.

[b]Total tons of NO_X reductions required includes 51,000 tons for Post-2025 California and 46,000 tons for the rest of the United States in 2025. Because these simulations are designed to be proof of concept, we combined the emissions reductions needed and controls applied in these analyses.

[c]Total tons of NO_X reductions required includes 100,000 tons for Post-2025 California and 860,000 tons for the rest of the United States in 2025. Because these simulations are designed to be proof of concept, we combined the emissions reductions needed and controls applied in these analyses.

Supplemental Table 6: Monetized Costs of Tons of NOx Emissions using EPA Final Ozone RIA 2015, (in billions 2011$).

	**Revised 65 ppb Standard Levels**			**Revised 70 ppb Standard Levels**		
	**EPA RIA**	**Lange et al. using EPA (2015) RIA**		**EPA RIA**	**Lange et al. using EPA (2015) RIA**	
		Minimum Cost of Initial Unidentified Methods is $15,000/ton	Minimum Cost of Initial Unidentified Methods is $19,000/ton		Minimum Cost of Initial Unidentified Methods is $15,000/ton	Minimum Cost of Initial Unidentified Methods is $19,000/ton
Identified Costs	$2.6	$2.6	$2.6	$0.7	$0.7	$0.7
Unidentified Costs	$12.6	$28.4	$31.1	$0.7	$0.8	$0.9
**Total Cost of Emission Reduction**	**$16**	**$31**	**$34**	**$1.4**	**$1.5**	**$1.6**

Note: All values are rounded to two significant figures. Unidentified control costs are based on an average cost-per-ton methodology described in chapter 4 of EPA Final Ozone RIA (2015).

For 65 ppb: Cost to mitigate 560,000 tons from identified sources and 860,000 tons from unidentified sources (non-CA).

For 70 ppb: Cost to mitigate 560,000 tons from identified sources and 46,000 tons from unidentified sources (non-CA).

.

Supplemental Table 7: Estimated Monetized Ozone and $PM_{2.5}$ Benefits for Revised and Alternative Annual Ozone Standards of 0.065 ppm using EPA 2015 Final Ozone RIA Table 6-24 as the basis (in billions of dollars) [a]

Revised 65 ppb Standard Levels			
	**EPA RIA (2015)**	**Lange et al. Alternative 1**	**Lange et al. Alternative 2**
Ozone-only benefits[b]	$5.3 to $8.7	$3.2 to $4.4	$0.3 to $0.5
$PM_{2.5}$ Cobenefits[c]	$9.3 to $21	$4.6 to $10	$0.6 to $1.3
**Total Benefits**	$15 to $30	$7.8 to $14	$0.9 to $1.9
**Identified Control Strategies Only**			
Ozone-only benefits[b]	$2.2 to $3.5	$1.3 to $1.8	$0.1 to $0.2
$PM_{2.5}$ Cobenefits[c]	$3.6 to $8.1	$1.8 to $4.9	$0.2 to $0.5
**Total Benefits**	$5.7 to $12	$3.1 to $6.7	$0.4 to $0.7

[a]Rounded to two significant figures. The reduction in premature fatalities each year accounts for over 98% of total monetized benefits in this analysis. Mortality risk valuation for $PM_{2.5}$ assumes discounting over the SAB-recommended 20-year segmented lag structure. These estimates reflect the economic value of avoided morbidities and premature deaths using risk coefficients from the studies noted

[b]Range reflects Smith et al, 2009 and Zanobetti & Schwartz, 2008. Ozone-only benefits reflect short-term exposure impacts and as such are assumed to occur in the same year as ambient ozone reductions. Consequently, social discounting is not applied to the benefits for this category

[c]Range reflects Krewski et al., 2009, and Lepeule et al., 2012. 7% discount rate.

Supplemental Table 8: Estimate of Monetized Ozone and $PM_{2.5}$ Net Benefits for Proposed Annual Ozone Standard of 0.065 ppm 2025 Scenario, using EPA Final Ozone RIA (2015) as the basis (nationwide benefits of attaining each alternative standard everywhere in the U.S. except California and except as noted) – Full Attainment (billions of 2011$) [a]

**Total Benefits** [a]		**Costs**			**Net Benefits**
**Source**		**Source**	**Minimum Cost of Initial Unidentified Method (per ton)**	**Total Costs**	
EPA (2015)	$15 to $30	EPA (2015) RIA	$15,000	$16	($1.0) to $14
		Lange et al. using EPA (2015) RIA	$15,000	$31	($16) to ($1.0)
		Lange et al. using EPA (2015) RIA	$19,000	$34	($19) to ($3.7)
Alternative 1	$7.8 to 14	EPA (2015) RIA	$15,000	$16	($8.2) to $2.0
		Lange et al. using EPA (2015) RIA	$15,000	$31	($23) to ($17)
		Lange et al. using EPA (2015) RIA	$19,000	$34	($26) to ($20)
Alternative 2	$0.9 to $1.9	EPA (2015) RIA	$15,000	$16	($15) to ($14)
		Lange et al. using EPA (2015) RIA	$15,000	$31	($30) to ($29)
		Lange et al. using EPA (2015) RIA	$19,000	$34	($33) to ($32)

[a]7% discount rate.
